# ***Barrmaelia*** and ***Entosordaria*** in Barrmaeliaceae (fam. nov., Xylariales) and critical notes on ***Anthostomella***-like genera based on multigene phylogenies

**DOI:** 10.1007/s11557-017-1329-6

**Published:** 2017-08-23

**Authors:** Hermann Voglmayr, Gernot Friebes, Alain Gardiennet, Walter M. Jaklitsch

**Affiliations:** 1 Division of Systematic and Evolutionary Botany, Department of Botany and Biodiversity Research, University of Vienna, Rennweg 14, 1030 Wien, Austria; 2 Centre of Natural History, Botany, Universalmuseum Joanneum, Weinzöttlstraße 16, 8045 Graz, Austria; 3 14 rue Roulette, 21260 Véronnes, France; 4 Institute of Forest Entomology, Forest Pathology and Forest Protection, Dept. of Forest and Soil Sciences, BOKU—University of Natural Resources and Life Sciences, Hasenauerstraße 38, 1190 Vienna, Austria

**Keywords:** *Anthostoma*, Ascomycota, *Clypeosphaeria*, Phylogenetic analysis, Pyrenomycetes, Sordariomycetes, *Stereosphaeria*, Xylariaceae

## Abstract

Phylogenetic analyses of a combined DNA data matrix containing ITS, LSU, *rpb2* and *tub2* sequences of representative Xylariales revealed that the genus *Barrmaelia* is a well-defined monophylum, as based on four of its described species (*B. macrospora*, *B. moravica*, *B. oxyacanthae*, *B. rhamnicola*) and the new species *B. rappazii*. The generic type of *Entosordaria*, *E. perfidiosa*, is revealed as the closest relative of *Barrmaelia*, being phylogenetically distant from the generic type of *Clypeosphaeria*, *C. mamillana*, which belongs to Xylariaceae sensu stricto. *Entosordaria* and *Barrmaelia* are highly supported and form a distinct lineage, which is recognised as the new family Barrmaeliaceae. The new species *E. quercina* is described. *Barrmaelia macrospora*, *B. moravica* and *B. rhamnicola* are epitypified and *E. perfidiosa* is lecto- and epitypified. Published sequences of *Anthostomella* and several *Anthostomella*-like species from the genera *Alloanthostomella*, *Anthostomelloides*, *Neoanthostomella*, *Pseudoanthostomella* and *Pyriformiascoma* are evaluated, demonstrating the necessity of critical inspection of published sequence data before inclusion in phylogenies. Verified isolates of several species from these genera should be re-sequenced to affirm their phylogenetic affinities. In addition, the generic type of *Anthostomella* should be sequenced before additional generic re-arrangements are proposed.

**Electronic supplementary material:**

The online version of this article (doi:10.1007/s11557-017-1329-6) contains supplementary material, which is available to authorized users.

## Introduction

Xylariaceae have long been treated in a conservative, morphology-based concept, and only informal subgroupings like Hypoxyloideae and Xylarioideae were accepted despite polyphyly of several genera. Recently, Wendt et al. (2017) subdivided Xylariaceae into three families based on multigene phylogeny of an ITS–LSU–*rpb2*–*tub2* matrix. They resurrected and emended the family Hypoxylaceae, widened the Graphostromataceae to include the genera *Biscogniauxia*, *Camillea*, *Obolarina* and *Vivantia*, and restricted Xylariaceae mostly to genera with geniculosporium-like asexual morphs. This facilitates phylogenetic placement of other genera affiliated with Xylariaceae sensu lato. One example is the genus *Anthostomella*, which houses a number of species, whose morphological traits vary considerably and may, thus, be phylogenetically uninformative. Ascomata are usually immersed in the host tissue, covered by a clypeus or not, have amyloid or sometimes non-amyloid ascus apices and brown amerosporous ascospores with or without a hyaline appendage cell, with or without a gelatinous sheath. One major challenge to study them on hosts other than palms is the difficulty to spot them, as they cannot be collected regularly, and, often, they produce very limited material. Francis (1975) performed a study on the systematics of *Anthostomella* species on the stems and leaves of herbaceous plants and gymnosperms based on morphology alone. A similar but more voluminous study was carried out by Lu and Hyde (2000). Using a few newly collected specimens, Daranagama et al. (2015, 2016) determined that *Anthostomella* is polyphyletic within Xylariaceae and described several new genera.

There is some confusion in the literature about the generic type of *Anthostomella*. Eriksson (1966) pointed out that lectotypification of *Anthostomella* with *A. phaeosticta* by Clements and Shear (1931) was in error and that *A. limitata* is the true generic type of *Anthostomella*. According to the ICN, this lectotypification is valid and has to be followed unless conservation with a different type is formally approved, and *A. limitata* is correctly listed as the generic type in Index Fungorum. In arguing that *A. limitata* does not exhibit several morphological characters then considered typical for the genus, Francis (1975) proposed *A. tomicoides* as the generic type, but this change has never been formally proposed and approved to become in effect. However, in the subsequent publications cited above, Francis (1975) was followed and *A. tomicoides* was accepted as the generic type. Neither *A. limitata* nor *A. tomicoides* have yet been sequenced.

Several genera have been segregated from *Anthostomella* or newly described, or subgenera were elevated to the generic rank. One of the latter is *Lopadostoma* (Jaklitsch et al. 2014) and another *Entosordaria*. The generic type of *Entosordaria*, *E. perfidiosa*, is characterised by non-amyloid asci and ascospores, which have a unique apical germ apparatus consisting of radiating slits (Eriksson 1966; Eriksson and Hawksworth 1986). Nonetheless, the genus was subsumed by Barr (1989) under *Clypeosphaeria* (see also Jaklitsch et al. 2016).

A transition to and now a member of the Diatrypaceae is the genus *Anthostoma*, which currently encompasses the single lignicolous species *A. decipiens* (Rappaz 1992; Jaklitsch et al. 2014). In a study designed to assess *Anthostomella* on hardwoods, Rappaz (1995) described the genus *Barrmaelia*, whose species, in part, also resemble Diatrypaceae, particularly in ascospore features, but, in contrast, have short-pedicellate asci and non-amyloid ascus apices. Furthermore, *Barrmaelia* species are typically characterised by ascomata that are immersed in the wood or bark and stromata that tend to blacken the host surface, in combination with light to dark brown, one-celled, smooth, ellipsoid to allantoid ascospores without sheath or appendages and with or without a germ slit. Rappaz (1995) combined six species in *Barrmaelia* (*B. macrospora*, *B. moravica*, *B. oxyacanthae*, *B. picacea*, *B. pseudobombarda* and *B. sustenta*) and described one new species, which he also selected as the generic type, *B. rhamnicola*. No new taxa have been added to this genus since then.

Although Rappaz (1995) only had morphology at hand, his concept withstands molecular phylogenetic analyses, as we show below. We, therefore, describe the new species *B. rappazii* to honour him, present the molecular systematics of five species of *Barrmaelia* and two of *Entosordaria*, including the new species *E. quercina*. The genera *Barrmaelia* and *Entosordaria* form a distinct lineage, which we name as the new family Barrmaeliaceae.

## Materials and methods

### Isolates and specimens

All newly prepared isolates used in this study originated from ascospores of fresh specimens. The numbers of strains including NCBI GenBank accession numbers of gene sequences used to compute the phylogenetic trees are listed in Table 1. Isolates have been deposited at the Westerdijk Fungal Biodiversity Institute (CBS-KNAW), Utrecht, the Netherlands. Details of the specimens used for morphological investigations are listed in the Taxonomy section under the respective descriptions. Herbarium acronyms are according to Thiers (2017). Specimens have been deposited in the Fungarium of the Institute of Botany, University of Vienna (WU).

**Table 1:** Isolates and accession numbers used in the phylogenetic analyses. Isolates/sequences in **bold** were isolated/sequenced in the present study. For details about sequence accessions retrieved from GenBank, see Jaklitsch and Voglmayr (2012), Jaklitsch et al. (2014, 2016), Daranagama et al. (2016), Hernández-Restrepo et al. (2016) and Wendt et al. (2017)

Species	Specimen or strain number^a^	Origin	Status^b^	GenBank accession numbers^c^				
				ITS	LSU	*rpb2*	*tub2*	*tef1*
*Amphirosellinia fushanensis*	HAST 91111209	Taiwan	HT	GU339496	N/A	GQ848339	GQ495950	
*Amphirosellinia nigrospora*	HAST 91092308	Taiwan	HT	GU322457	N/A	GQ848340	GQ495951	
*Annulohypoxylon annulatum*	CBS 140775	Texas	ET	KY610418	KY610418	KY624263	KX376353	
*Annulohypoxylon atroroseum*	ATCC 76081	Thailand		AJ390397	KY610422	KY624233	DQ840083	
*Annulohypoxylon michelianum*	CBS 119993	Spain		KX376320	KY610423	KY624234	KX271239	
*Annulohypoxylon moriforme*	CBS 123579	Martinique		KX376321	KY610425	KY624289	KX271261	
*Annulohypoxylon nitens*	MFLUCC 12-0823	Thailand		KJ934991	KJ934992	KJ934994	KJ934993	
*Annulohypoxylon stygium*	MUCL 54601	French Guiana		KY610409	KY610475	KY624292	KX271263	
*Annulohypoxylon truncatum*	CBS 140778	Texas	ET	KY610419	KY610419	KY624277	KX376352	
*Anthostomella formosa*	MFLUCC 14-0170	Italy		KP297403	KP340544	KP340531	N/A^d^	
*Anthostomella helicofissa*	MFLUCC 14-0173	Italy	HT	KP297406	KP297406^e^	KP340534	KP406617	
*Anthostomella obesa*	MFLUCC 14-0171	Italy	HT	KP297405	KP340546	KP340533	N/A^d^	
*Anthostomella rubicola*	MFLUCC 16-0479	Italy		KX533455	KX533456	KX789493	KX789494	
*Anthostomelloides forlicesenica*	MFLUCC 14-0007	Italy	HT	KP297396	KP297396^e,f^	N/A^f^	KP406607	
*Anthostomelloides krabiensis*	MFLUCC 15-0678	Thailand	HT	KX305927	KX305928	KX305929	N/A	
*Astrocystis concavispora*	MFLUCC 14-0174	Italy		KP297404	KP340545	KP340532	KP406615	
*Barrmaelia macrospora*	**BM = CBS 142768**	Austria	ET	KC774566	KC774566	**MF488995**	**MF489014**	**MF489005**
*Barrmaelia moravica*	**Cr1 = CBS 142769**	Austria	ET	**MF488987**	**MF488987**	**MF488996**	**MF489015**	**MF489006**
*Barrmaelia oxyacanthae*	**BO = CBS 142770**	Austria		**MF488988**	**MF488988**	**MF488997**	**MF489016**	**MF489007**
*Barrmaelia rappazii*	**Cr2 = CBS 142771**	Norway	HT	**MF488989**	**MF488989**	**MF488998**	**MF489017**	**MF489008**
*Barrmaelia rhamnicola*	**BR = CBS 142772**	France	ET	**MF488990**	**MF488990**	**MF488999**	**MF489018**	**MF489009**
*Barrmaelia rhamnicola*	**BR1**	France		**MF488991**	**MF488991**	**MF489000**	**MF489019**	**MF489010**
*Biscogniauxia arima*	WSP 122	Mexico	IT	EF026150	N/A	GQ304736	AY951672	
*Biscogniauxia atropunctata*	Y.M.J. 128	USA		JX507799	N/A	JX507778	AY951673	
*Biscogniauxia marginata*	MFLUCC 12-0740	France		KJ958407	KJ958408	KJ958409	KJ958406	
*Biscogniauxia nummularia*	MUCL 51395	France	ET	KY610382	KY610427	KY624236	KX271241	
*Biscogniauxia repanda*	ATCC 62606	USA		KY610383	KY610428	KY624237	KX271242	
*Brunneiperidium gracilentum*	MFLUCC 14-0011	Italy	HT	KP297400	KP340542	KP340528	KP406611	
*Calceomyces lacunosus*	CBS 633.88	Japan	HT	KY610397	KY610476	KY624293	KX271265	
*Camillea obularia*	ATCC 28093	Puerto Rico		KY610384	KY610429	KY624238	KX271243	
*Camillea tinctor*	Y.M.J. 363	Martinique		JX507806	N/A	JX507790	JX507795	
*Clypeosphaeria mamillana*	CLM = CBS 140735	France	ET	KT949897	KT949897	**MF489001**	N/A	
*Collodiscula bambusae*	GZUH 0102	China		KP054279	KP054280	KP276675	KP276674	
*Collodiscula fangjingshanensis*	GZUH 0109	China	HT	KR002590	KR002591	KR002592	KR002589	
*Collodiscula japonica*	CBS 124266	China		JF440974	JF440974	KY624273	KY624316	
*Creosphaeria sassafras*	ST.MA. 14087	Argentina		KY610411	KY610468	KY624265	KX271258	
*Daldinia andina*	CBS 114736	Ecuador	HT	AM749918	KY610430	KY624239	KC977259	
*Daldinia bambusicola*	CBS 122872	Thailand	HT	KY610385	KY610431	KY624241	AY951688	
*Daldinia caldariorum*	MUCL 49211	France		AM749934	KY610433	KY624242	KC977282	
*Daldinia concentrica*	CBS 113277	Germany		AY616683	KY610434	KY624243	KC977274	
*Daldinia dennisii*	CBS 114741	Australia	HT	JX658477	KY610435	KY624244	KC977262	
*Daldinia eschscholtzii*	MUCL 45435	Benin		JX658484	KY610437	KY624246	KC977266	
*Daldinia loculatoides*	CBS 113279	UK	ET	AF176982	KY610438	KY624247	KX271246	
*Daldinia macaronesica*	CBS 113040	Spain	PT	KY610398	KY610477	KY624294	KX271266	
*Daldinia petriniae*	MUCL 49214	Austria	ET	AM749937	KY610439	KY624248	KC977261	
*Daldinia placentiformis*	MUCL 47603	Mexico		AM749921	KY610440	KY624249	KC977278	
*Daldinia pyrenaica*	MUCL 53969	France		KY610413	KY610413	KY624274	KY624312	
*Daldinia steglichii*	MUCL 43512	Papua New Guinea	PT	KY610399	KY610479	KY624250	KX271269	
*Daldinia theissenii*	CBS 113044	Argentina	PT	KY610388	KY610441	KY624251	KX271247	
*Daldinia vernicosa*	CBS 119316	Germany	ET	KY610395	KY610442	KY624252	KC977260	
*Diatrype disciformis*	CBS 197.49	Netherlands		N/A	DQ470964	DQ470915	N/A	
*Entoleuca mammata*	J.D.R. 100	France		GU300072	N/A	GQ844782	GQ470230	
*Entonaema liquescens*	ATCC 46302	USA		KY610389	KY610443	KY624253	KX271248	
*Entosordaria perfidiosa*	**BW3**	Germany		**MF488992**	**MF488992**	**MF489002**	**MF489020**	**MF489011**
*Entosordaria perfidiosa*	**EPE = CBS 142773**	Austria	ET	**MF488993**	**MF488993**	**MF489003**	**MF489021**	**MF489012**
*Entosordaria quercina*	**RQ = CBS 142774**	Greece	HT	**MF488994**	**MF488994**	**MF489004**	**MF489022**	**MF489013**
*Euepixylon sphaeriostomum*	J.D.R. 261	USA		GU292821	N/A	GQ844774	GQ470224	
*Eutypa lata*	UCR-EL1	USA		JGI	JGI	JGI	JGI	
*Graphostroma platystomum*	CBS 270.87	France	HT	JX658535	DQ836906	KY624296	HG934108	
*Hypocreodendron sanguineum*	J.D.R. 169	Mexico		GU322433	N/A	GQ844819	GQ487710	
*Hypoxylon carneum*	MUCL 54177	France		KY610400	KY610480	KY624297	KX271270	
*Hypoxylon cercidicola*	CBS 119009	France		KC968908	KY610444	KY624254	KC977263	
*Hypoxylon crocopeplum*	CBS 119004	France		KC968907	KY610445	KY624255	KC977268	
*Hypoxylon fendleri*	MUCL 54792	French Guiana		KF234421	KY610481	KY624298	KF300547	
*Hypoxylon fragiforme*	MUCL 51264	Germany	ET	KC477229	KM186295	KM186296	KX271282	
*Hypoxylon fuscum*	CBS 113049	France	ET	KY610401	KY610482	KY624299	KX271271	
*Hypoxylon griseobrunneum*	CBS 331.73	India	HT	KY610402	KY610483	KY624300	KC977303	
*Hypoxylon haematostroma*	MUCL 53301	Martinique	ET	KC968911	KY610484	KY624301	KC977291	
*Hypoxylon howeanum*	MUCL 47599	Germany		AM749928	KY610448	KY624258	KC977277	
*Hypoxylon hypomiltum*	MUCL 51845	Guadeloupe		KY610403	KY610449	KY624302	KX271249	
*Hypoxylon investiens*	CBS 118183	Malaysia		KC968925	KY610450	KY624259	KC977270	
*Hypoxylon lateripigmentum*	MUCL 53304	Martinique	HT	KC968933	KY610486	KY624304	KC977290	
*Hypoxylon lenormandii*	CBS 119003	Ecuador		KC968943	KY610452	KY624261	KC977273	
*Hypoxylon monticulosum*	MUCL 54604	French Guiana	ET	KY610404	KY610487	KY624305	KX271273	
*Hypoxylon musceum*	MUCL 53765	Guadeloupe		KC968926	KY610488	KY624306	KC977280	
*Hypoxylon ochraceum*	MUCL 54625	Martinique	ET	KC968937	N/A	KY624271	KC977300	
*Hypoxylon papillatum*	ATCC 58729	USA	HT	KC968919	KY610454	KY624223	KC977258	
*Hypoxylon perforatum*	CBS 115281	France		KY610391	KY610455	KY624224	KX271250	
*Hypoxylon petriniae*	CBS 114746	France	HT	KY610405	KY610491	KY624279	KX271274	
*Hypoxylon pilgerianum*	ST.MA. 13455	Martinique		KY610412	KY610412	KY624308	KY624315	
*Hypoxylon porphyreum*	CBS 119022	France		KC968921	KY610456	KY624225	KC977264	
*Hypoxylon pulicicidum*	CBS 122622	Martinique	HT	JX183075	KY610492	KY624280	JX183072	
*Hypoxylon rickii*	MUCL 53309	Martinique	ET	KC968932	KY610416	KY624281	KC977288	
*Hypoxylon rubiginosum*	MUCL 52887	Germany	ET	KC477232	KY610469	KY624266	KY624311	
*Hypoxylon samuelsii*	MUCL 51843	Guadeloupe	ET	KC968916	KY610466	KY624269	KC977286	
*Hypoxylon submonticulosum*	CBS 115280	France		KC968923	KY610457	KY624226	KC977267	
*Hypoxylon ticinense*	CBS 115271	France		JQ009317	KY610471	KY624272	AY951757	
*Hypoxylon trugodes*	MUCL 54794	Sri Lanka	ET	KF234422	KY610493	KY624282	KF300548	
*Hypoxylon vogesiacum*	CBS 115273	France		KC968920	KY610417	KY624283	KX271275	
*Jackrogersella cohaerens*	CBS 119126	Germany		KY610396	KY610497	KY624270	KY624314	
*Jackrogersella minutella*	CBS 119015	Portugal		KY610381	KY610424	KY624235	KX271240	
*Jackrogersella multiformis*	CBS 119016	Germany	ET	KC477234	KY610473	KY624290	KX271262	
*Kretzschmaria deusta*	CBS 163.93	Germany		KC477237	KY610458	KY624227	KX271251	
*Lopadostoma dryophilum*	CBS 133213	Austria	ET	KC774570	KC774570	KC774526	**MF489023**	
*Lopadostoma turgidum*	CBS 133207	Austria	ET	KC774618	KC774618	KC774563	**MF489024**	
*Microdochium lycopodinum*	CBS 122885	Austria	HT	JF440979	JF440979	KP859125	KP859080	
*Microdochium phragmitis*	CBS 285.71	Poland	ET	KP859013	KP858949	KP859122	KP859077	
*Microdochium seminicola*	CBS 139951	Switzerland	HT	KP859038	KP858974	KP859147	KP859101	
*Nemania abortiva*	BISH 467	USA	HT	GU292816	N/A	GQ844768	GQ470219	
*Nemania beaumontii*	HAST 405	Martinique		GU292819	N/A	GQ844772	GQ470222	
*Nemania bipapillata*	HAST 90080610	Taiwan		GU292818	N/A	GQ844771	GQ470221	
*Nemania maritima*	HAST 89120401	Taiwan	ET	N/A	N/A	GQ844775	GQ470225	
*Nemania maritima*	ST.MA. 04019 = J.F. 03075	France		KY610414	KY610414	N/A	N/A	
*Nemania primolutea*	HAST 91102001	Taiwan	HT	EF026121	N/A	GQ844767	EF025607	
*Neoanthostomella viticola*	MFLUCC 16-0243	Italy	HT	KX505957	KX505958	KX789496	KX789495	
*Obolarina dryophila*	MUCL 49882	France		GQ428316	GQ428316	KY624284	GQ428322	
*Podosordaria mexicana*	WSP 176	Mexico		GU324762	N/A	GQ853039	GQ844840	
*Podosordaria muli*	WSP 167	Mexico	HT	GU324761	N/A	GQ853038	GQ844839	
*Poronia pileiformis*	WSP 88113001	Taiwan	ET	GU324760	N/A	GQ853037	GQ502720	
*Poronia punctata*	CBS 656.78	Australia	HT	KT281904	KY610496	KY624278	KX271281	
*Pseudoanthostomella delitescens*	MFLUCC 16-0477	Italy		KX533451	KX533452	KX789491	KX789490	
*Pseudoanthostomella pini-nigrae*	MFLUCC 16-0478	Italy	HT	KX533453	KX533454	KX789492	N/A	
*Pseudoanthostomella senecionicola*	MFLUCC 15-0013	Italy	HT	KX505960	KX505959	KX789489	N/A	
*Pyrenopolyporus hunteri*	MUCL 52673	Ivory Coast	ET	KY610421	KY610472	KY624309	KU159530	
*Pyrenopolyporus laminosus*	MUCL 53305	Martinique	HT	KC968934	KY610485	KY624303	KC977292	
*Pyrenopolyporus nicaraguensis*	CBS 117739	Burkina Faso		AM749922	KY610489	KY624307	KC977272	
*Pyriformiascoma trilobatum*	MFLUCC 14-0012	Italy	HT	KP297402	KP340543	KP340530	KP406613	
*Rhopalostroma angolense*	CBS 126414	Ivory Coast		KY610420	KY610459	KY624228	KX271277	
*Rosellinia aquila*	MUCL 51703	France		KY610392	KY610460	KY624285	KX271253	
*Rosellinia buxi*	J.D.R. 99	France		GU300070	N/A	GQ844780	GQ470228	
*Rosellinia corticium*	MUCL 51693	France		KY610393	KY610461	KY624229	KX271254	
*Rosellinia necatrix*	CBS 349.36	Argentina		AY909001	KF719204	KY624275	KY624310	
*Rostrohypoxylon terebratum*	CBS 119137	Thailand	HT	DQ631943	DQ840069	DQ631954	DQ840097	
*Ruwenzoria pseudoannulata*	MUCL 51394	D. R. Congo	HT	KY610406	KY610494	KY624286	KX271278	
*Sarcoxylon compunctum*	CBS 359.61	South Africa		KT281903	KY610462	KY624230	KX271255	
*Stilbohypoxylon elaeicola*	Y.M.J. 173	French Guiana		EF026148	N/A	GQ844826	EF025616	
*Stilbohypoxylon quisquiliarum*	Y.M.J. 172	French Guiana		EF026119	N/A	GQ853020	EF025605	
*Thamnomyces dendroidea*	CBS 123578	French Guiana	HT	FN428831	KY610467	KY624232	KY624313	
*Xylaria acuminatilongissima*	HAST 95060506	Taiwan	HT	EU178738	N/A	GQ853028	GQ502711	
*Xylaria adscendens*	J.D.R. 865	Thailand		GU322432	N/A	GQ844818	GQ487709	
*Xylaria arbuscula*	CBS 126415	Germany		KY610394	KY610463	KY624287	KX271257	
*Xylaria bambusicola*	WSP 205	Taiwan	HT	EF026123	N/A	GQ844802	AY951762	
*Xylaria brunneovinosa*	HAST 720	Martinique	HT	EU179862	N/A	GQ853023	GQ502706	
*Xylaria curta*	HAST 494	Martinique		GU322444	N/A	GQ844831	GQ495937	
*Xylaria discolour*	HAST 131023	USA	ET	JQ087405	N/A	JQ087411	JQ087414	
*Xylaria hypoxylon*	CBS 122620	Sweden	ET	KY610407	KY610495	KY624231	KX271279	
*Xylaria multiplex*	HAST 580	Martinique		GU300098	N/A	GQ844814	GQ487705	
*Xylaria polymorpha*	MUCL 49884	France		KY610408	KY610464	KY624288	KX271280	

^a^ATCC, American Type Culture Collection, Manassas, USA; BISH, Bishop Museum, Honolulu, USA; CBS, Westerdijk Fungal Biodiversity Institute, Utrecht, the Netherlands; GZUH, Guizhou University, Guiyang, China; HAST, Academia Sinica, Taipei, Taiwan; J.D.R., Jack D. Rogers, Washington State University, Pullman, USA; J.F., Jacques Fournier, Rimont, France; MFLUCC, Mae Fah Luang University, Chiang Rai, Thailand; MUCL, Université Catholique de Louvain, Louvain-la-Neuve, Belgium; ST.MA., Marc Stadler, Helmholtz-Zentrum für Infektionsforschung, Braunschweig, Germany; UCR, University of California, Riverside, USA; Y.M.J., Yu-Ming Ju, Academia Sinica, Taipei, Taiwan; WSP, Washington State University, Pullman, USA

^b^ET, epitype; HT, holotype; IT, isotype; PT, paratype

^c^N/A, not available; JGI, sequences retrieved from JGI-DOE (http://genome.jgi.doe.gov/)

^d^ *tub2* sequences of Daranagama et al. (2015) not included, as they are erroneous, actually representing *rpb2* sequences of an unidentified fungus

^e^Partial LSU of the deposited ITS sequences used for analyses, as the LSU sequences of Daranagama et al. (2015) are not from the same fungus (highly distinct from the LSU part of the ITS)

^f^GenBank sequences of LSU and *rpb2* of Daranagama et al. (2015) not included, as they are erroneous, representing sequences of an unidentified pleosporalean fungus

### Culture preparation, growth rate determination and phenotype analysis

Cultures were prepared and maintained as described previously (Jaklitsch 2009). Microscopic observations were made in tap water, except where noted. Morphological analyses of microscopic characters were carried out as described earlier (Jaklitsch 2009). Methods of microscopy included stereomicroscopy using Nikon SMZ1500, Olympus SZX10 and Euromex Novex RZ 65.560, light microscopy using Euromex XHR MIC 625, Olympus BX51 and Nomarski differential interference contrast (DIC) using the compound microscopes Nikon Eclipse E600 and Zeiss Axio Imager.A1. Images and data were gathered with Nikon Coolpix 4500, Nikon DS-U2, Nikon D90, Olympus DP72 and Zeiss Axiocam 506 colour digital cameras and measured directly with the microscope, or with Olympus cellSens Dimension, NIS-Elements D v.3.0 and Zeiss ZEN Blue Edition softwares. Amyloidity of asci was assessed using Lugol or Melzer reagent. Measurements are reported as maximum and minimum in parentheses and the range representing the mean plus and minus the standard deviation of a number of measurements given in parentheses.

### DNA extraction and sequencing methods

The extraction of genomic DNA was performed as reported previously (Voglmayr and Jaklitsch 2011; Jaklitsch et al. 2012) using the DNeasy Plant Mini Kit (QIAgen GmbH, Hilden, Germany). The following loci were amplified and sequenced: the complete internally transcribed spacer region (ITS1–5.8S–ITS2) and a ca. 1.3-kb fragment of the large subunit nuclear ribosomal DNA (nLSU rDNA), amplified and sequenced as a single fragment with primers V9G (de Hoog and Gerrits van den Ende 1998) and LR5 (Vilgalys and Hester 1990); a ca. 1.2-kb fragment of the RNA polymerase II subunit 2 (*rpb2*) gene with primers fRPB2-5f and fRPB2-7cr (Liu et al. 1999) or dRPB2-5f and dRPB2-7r (Voglmayr et al. 2016a); a ca. 1.3–1.5-kb fragment of the translation elongation factor 1-alpha (*tef1*) gene with primers EF1-728F (Carbone and Kohn 1999) and TEF1LLErev (Jaklitsch et al. 2005) or EF1-2218R (Rehner and Buckley 2005); and a ca. 1.6-kb fragment of the beta-tubulin (*tub2*) gene with primers T1 and T22 (O’Donnell and Cigelnik 1997). Polymerase chain reaction (PCR) products were purified using an enzymatic PCR cleanup (Werle et al. 1994) as described in Voglmayr and Jaklitsch (2008). DNA was cycle-sequenced using the ABI PRISM BigDye Terminator Cycle Sequencing Ready Reaction Kit v.3.1 (Applied Biosystems, Warrington, UK) with the same primers as in PCR; in addition, primers ITS4 (White et al. 1990), LR2R-A (Voglmayr et al. 2012) and LR3 (Vilgalys and Hester 1990) were used for the ITS–LSU region, TEF1_INTF (Jaklitsch 2009) and TEFD_iR (5′ GTCTGGCCATCCTTGGAGAT 3′) for *tef1* and BtHV2r (Voglmayr et al. 2016b, 2017) for *tub2*. Sequencing was performed on an automated DNA sequencer (3730xl Genetic Analyser, Applied Biosystems).

### Analysis of sequence data

Following the phylogenetic placement of *Barrmaelia macrospora* within the Xylariaceae sensu lato clade in earlier analyses (Jaklitsch et al. 2014, 2016), sequences of *Barrmaelia* and *Entosordaria* were analysed within the combined ITS, LSU rDNA, *rpb2* and *tub2* matrix of Wendt et al. (2017). As only a few *tef1* sequences are available for Xylariales, this marker was not included in the matrix but the sequences were deposited at GenBank as a secondary barcode marker. To obtain a more representative taxon sampling, selected sequences were added to this matrix from Hernández-Restrepo et al. (2016) and from Daranagama et al. (2015, 2016). From the latter two publications dealing with *Anthostomella*-like representatives, only accessions for which at least three of the four loci are available were included; before addition, it proved necessary to check these sequences with NCBI nucleotide BLAST searches for their correct gene and lineage identity, and obviously erroneous sequences as well as regions of poor sequence quality were excluded. For *Eutypa lata*, sequences were retrieved from the genome of strain UCR-EL1 deposited at JGI-DOE (http://genome.jgi.doe.gov/). Following the analyses of Jaklitsch et al. (2016), sequences of *Microdochium* (Microdochiaceae) were selected as the outgroup to root the trees. Familial classification of Xylariaceae sensu lato follows Wendt et al. (2017). All alignments were produced with the server version of MAFFT (http://www.ebi.ac.uk/Tools/msa/mafft), checked and refined using BioEdit version 7.0.9.0 (Hall 1999). After exclusion of ambiguously aligned regions and long gaps, the final matrix contained 4668 nucleotide characters, i.e. 600 from the ITS, 1359 from the LSU, 1162 from *rpb2* and 1547 from *tub2*.

Maximum parsimony (MP) analysis of the combined matrix was performed using a parsimony ratchet approach. For this, a nexus file was prepared using PRAP v.2.0b3 (Müller 2004), implementing 1000 ratchet replicates with 25% of randomly chosen positions upweighted to 2, which was then run with PAUP v.4.0a151 (Swofford 2002). The resulting best trees were then loaded in PAUP and subjected to heuristic search with TBR branch swapping (MULTREES option in effect, steepest descent option not in effect). Bootstrap analysis with 1000 replicates was performed using five rounds of replicates of heuristic search with random addition of sequences and subsequent TBR branch swapping (MULTREES option in effect, steepest descent option not in effect) during each bootstrap replicate. In all MP analyses, molecular characters were unordered and given equal weight; analyses were performed with gaps treated as missing data; the COLLAPSE command was set to minbrlen.

Maximum likelihood (ML) analyses were performed with RAxML (Stamatakis 2006) as implemented in raxmlGUI 1.3 (Silvestro and Michalak 2012), using the ML + rapid bootstrap setting and the GTRGAMMA substitution model with 1000 bootstrap replicates. The matrix was partitioned for the individual gene regions, and substitution model parameters were calculated separately for them.

## Results

### Assessment of published sequences

NCBI Nucleotide BLAST searches revealed serious problems for some sequences of Daranagama et al. (2015), which were, therefore, excluded from the analyses (Table 1). LSU sequences KP340547 (*Anthostomella helicofissa*) and KP340538 (*Anthostomelloides forlicesenica*) were not added to the matrix, as they did not correspond to the LSU part (ca. 540 bp) included in the ITS sequences KP297406 and KP297396 of the same accessions. Whereas LSU sequence KP340547 was revealed as xylarialean by BLAST searches but differed in 60 positions (3 gaps and 57 substitutions) from the LSU part of KP297406, a BLAST search of KP340538 revealed various Pleosporales (*Kalmusia*, *Coniothyrium*, *Dendrothyrium*) as the closest match (84% sequence similarity). Therefore, for these two accessions, only the LSU part of the ITS sequences was included in the LSU matrix. *rpb2* sequence KP340524 (*Anthostomelloides forlicesenica*) was excluded as well, as a BLAST search also revealed pleosporalean affinities (80% similarity to sequence LK936413 of *Leptosphaerulina chartarum*, 77% similarity to sequences DQ677970 of *Phaeodothis winteri* and DQ677956 of *Coniothyrium palmarum*). *tub2* sequences KP406614 (*Anthostomella formosa*) and KP406616 (*Anthostomella obesa*) were also excluded, as BLAST searches actually revealed them as *rpb2* sequences. This was also confirmed in an alignment containing the *rpb2* sequences included in the present study, where they were highly similar to *rpb2* sequences of various *Anthostomella* species (not shown); however, both were different from the *rpb2* sequences KP340531 and KP340533 published for the same isolates in the same publication.

### Molecular phylogeny

**Fig. 1:**
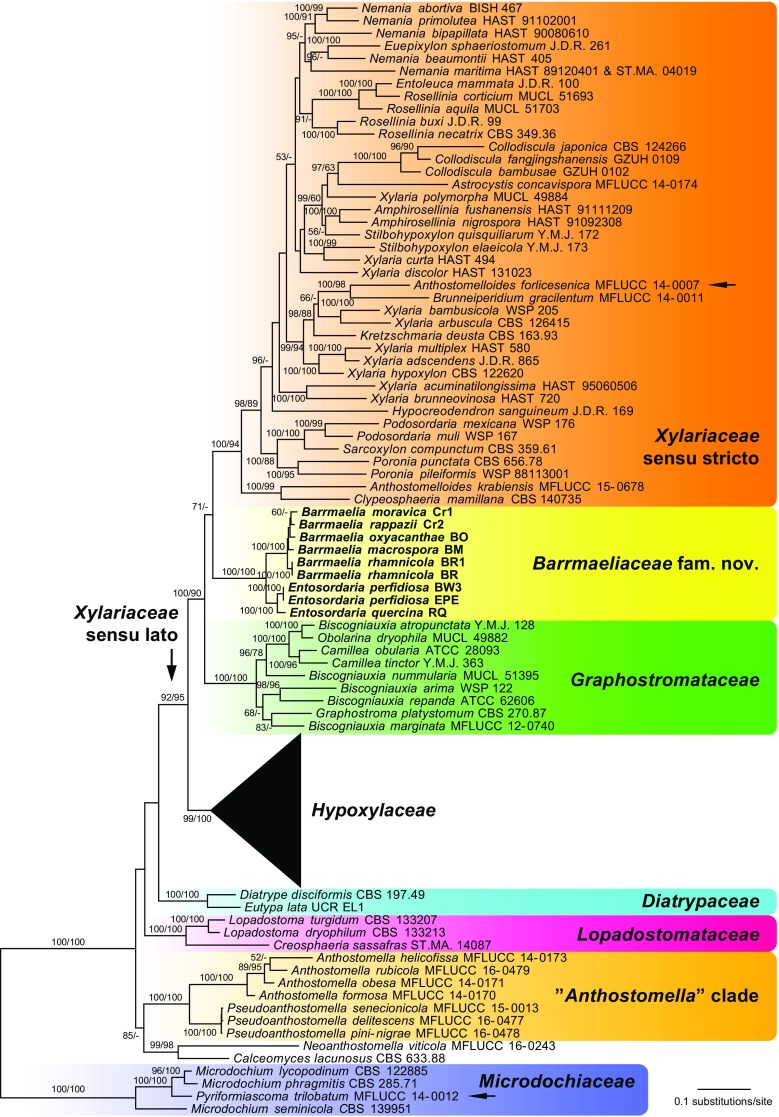
Simplified phylogram of the best ML trees (lnL = −136212.706) revealed by RAxML from an analysis of the combined ITS–LSU–*rpb2*–*tub2* matrix of selected Xylariales. Strains in **bold** were sequenced in the current study. The Hypoxylaceae clade, which is not treated in detail, is collapsed to provide sufficient space for the other clades of interest. ML and MP bootstrap support above 50% are given at the first and second positions, respectively, above or below the branches. The *arrows* denote topological conflict with previous phylogenies (*Anthostomelloides forlicesenica*) or major incongruence with the morphology of the clade in which it is placed (*Pyriformiascoma trilobatum*)

Of the 4668 nucleotide characters of the combined matrix, 2210 are parsimony informative (338 of ITS, 422 of LSU, 638 of *rpb2* and 812 of *tub2*). Figure 1 shows a simplified phylogram of the best ML tree (lnL = −136212.706) obtained by RAxML. Maximum parsimony analyses revealed four MP trees 32,311 steps long, which were identical except for a polytomy within the three terminal taxa of *Anthostomella* and an unresolved position of *Hypoxylon ochraceum* and *H. pilgerianum* relative to each other; the strict consensus tree of the four MP trees is provided in the Supplementary Information. The backbone of the MP trees was similar to the ML tree, except for a sister group relationship of Lopadostomataceae and Diatrypaceae and a slightly different position of the *Calceomyces*–*Neoanthostomella* clade; in addition, there were a few minor topological differences within the Xylariaceae and Graphostromataceae.

All families received high to maximum support in all analyses, as did the Xylariaceae sensu lato (Fig. 1). The genera *Barrmaelia* and *Entosordaria* were revealed as the closest relatives with maximum support but formed a separate lineage within Xylariaceae sensu lato, and are classified here within the new family Barrmaeliaceae. Within the Xylariaceae sensu lato, the basal position of Hypoxylaceae was highly supported, but the phylogenetic relationships between the other three families (Barrmaeliaceae, Graphostromataceae and Xylariaceae sensu stricto) remain uncertain due to the lack of significant backbone support. *Clypeosphaeria mamillana* is revealed as the closest relative of *Anthostomelloides krabiensis* with high (99% MP BS) to maximum (ML) support, and both are sister clade to the rest of the Xylariaceae sensu stricto with high support as well (Fig. 1). The second species of *Anthostomelloides*, *A. forlicesenica*, is not closely related to *A. krabiensis* but sister species of *Brunneiperidium gracilentum* within Xylariaceae sensu stricto with high (98% MP BS) to maximum (ML) support. The genera *Anthostomella* and *Pseudoanthostomella* are placed outside Xylariaceae sensu lato and form a highly supported lineage; sister group relationship to the highly supported *Calceomyces*–*Neoanthostomella* clade is revealed with medium support only in the ML analyses. *Pyriformiascoma trilobatum* is placed within *Microdochium* with maximum support in both analyses.

### Taxonomy

**Barrmaeliaceae** Voglmayr & Jaklitsch, fam. nov.

MycoBank MB 822042

*Type genus*: *Barrmaelia* Rappaz.

*Other genus in the family*: *Entosordaria* Höhn.

Saprobic on wood or bark. Stroma if present mostly in wood and blackening the surface in wide areas or in elongate bands, sometimes darker around the ostioles; entostroma prosenchymatous, poorly developed, without KOH-extractable pigments. Ascomata (perithecia) globose, sometimes raising the substrate, singly, in small groups or gregarious. Peridium melanised, pseudoparenchymatous to prosenchymatous. Hamathecium of numerous persistent, hyaline, septate paraphyses. Asci eight-spored, cylindrical, persistent, with inamyloid or infrequently amyloid apical ascus apparatus. Ascospores yellow to dark brown; unicellular with or without germ slit (*Barrmaelia*), or two-celled with septum near one end, the small cell hyaline, the large cell dark brown and with an apical germ apparatus consisting of radial slits (*Entosordaria*); allantoid or ellipsoid, inequilateral, slightly inequilateral or nearly equilateral, with narrowly or broadly rounded ends. Asexual morph libertella-like where known (*Barrmaelia*; Rappaz 1995).

***Barrmaelia*** Rappaz, Mycol. Helv. 7(1): 130 (1995).

*Type species*: *Barrmaelia rhamnicola* Rappaz, Mycol. Helv. 7(1): 131 (1995).

Stromata mostly in wood, usually discolouring the wood surface grey to black, entostroma poorly developed (Rappaz 1995). Ascomata perithecial, immersed in wood or bark, rarely erumpent, often blackening the host surface, globose, ellipsoid or pyriform; ostiolar pore rounded. Peridium melanised, pseudoparenchymatous to prosenchymatous. Hamathecium of apically free, hyaline paraphyses. Asci unitunicate, cylindrical, with a short stipe, generally eight-spored; with an inamyloid apical apparatus. Ascospores light to dark brown, one-celled, smooth, asymmetrically ellipsoid to allantoid, without sheath or appendages, with or without a germ slit. Asexual morph (fide Rappaz 1995) libertella-like, only known from pure culture, conidiomata globose, more or less melanised, up to 1 mm in diam., exuding the conidia in white to pinkish droplets. Conidiophores erect, branched. Conidiogenous cells hyaline, conical or cylindrical, arranged in palisades, apex sometimes with faint annellations. Conidiogenesis holoblastic, proliferation percurrent or sympodial. Conidia hyaline, falcate, one end truncate, the other rounded or slightly acute.

*Notes*: As we did not observe an asexual morph in pure culture, its description is adapted from Rappaz (1995).

**Fig. 2:**
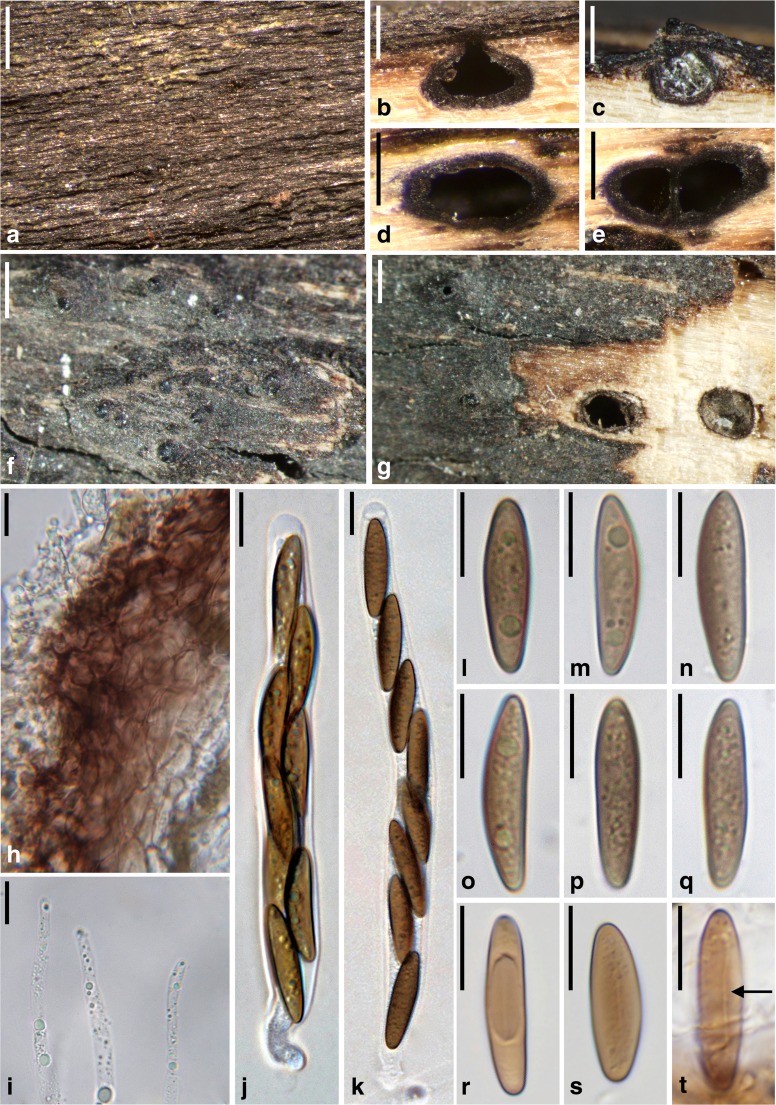
*Barrmaelia macrospora* (**a**, **b**, **d**, **e**, **h**–**q**: WU 36920, epitype; **c**, **f**, **g**, **r**–**t**: B 70 0009349). **a** Stroma with blackened wood surface. **b**, **c** Perithecia in vertical section. **d**, **e** Perithecia in transverse section. **f**, **g** Ostioles protruding through the blackened wood surface (**g** with perithecia in transverse section). **h** Vertical section of perithecial wall. **i** Paraphyses apices. **j**, **k** Asci. **l**–**t** Ascospores; arrow denoting germ slit (**t**). All in water. Scale bars: **a**, **c**, **f**, **g** = 500 μm; **b**, **d**, **e** = 250 μm; **h**, **i** = 5 μm; **j**–**t** = 10 μm

***Barrmaelia macrospora*** (Nitschke) Rappaz, Mycol. Helv. 7(1): 135 (1995). Fig. 2.

*Basionym*. *Valsa macrospora* Nitschke, Pyrenomyc. Germ. 1: 145 (1867).

For synonyms, see Rappaz (1995).

Stromata blackening the wood surface in areas of up to 5 × 1.5 cm. Wood usually unchanged among ascomata, sometimes slightly pale brown. Ascomata perithecial, 400–600 μm diam., 300–500 μm high (*n* = 10), usually gregarious but separate, rarely two in contact, immersed, depressed globose to ellipsoid. Ostiolar apices inconspicuous, sometimes slightly raised, circular. Peridium 20–35 μm thick (*n* = 10), pseudoparenchymatous at the outer side and consisting of moderately thick-walled cells encrusted with brown material, tending to be prosenchymatous, lighter coloured and thinner-walled at the inner side, partly filled with oil drops. Paraphyses numerous, filled with oil drops, 2–4 μm wide, slightly tapering towards the apex, obtuse. Asci 108–143 × 9–11 μm, spore part 91–123 μm long, stipe 5–21 μm long (*n* = 20), cylindrical, containing eight biseriate or obliquely uniseriate ascospores, with short stipe and an inamyloid apical apparatus. Ascospores (18.2–)20.5–24.0(−26.0) × (4.0–)4.8–5.9(−6.5) μm, l/w = (3.1–)3.7–4.7(−5.4) μm (*n* = 60), one-celled, narrowly ellipsoid to fusoid, asymmetric, ends sometimes slightly pointed, brown, germ slit hard to observe, with a lighter coloured band at the concave side, apically also sometimes lighter coloured, filled with minute oil drops, smooth.

Colonies on CMD and MEA white; aerial hyphae abundant. No asexual morph observed.

*Habitat*: In wood of (partly) decorticated twigs and branches of *Populus* spp., also on *Ligustrum* (fide Rappaz 1995).

*Distribution*: Europe (Czech Republic, France, Germany, Netherlands, Norway, Sweden, Switzerland, United Kingdom), possibly also the USA (fide Rappaz 1995).

*Typification*. Germany, Nordrhein-Westfalen, Münsterland, [Münster-] Handorf, on *Sarothamnus scoparius*, without date, Th. Nitschke, (B 70 0009297; sub *Valsa macrospora*, holotype, labelled as “Lectotype”). Epitype of *Valsa macrospora*, here designated: France, Côte-d’Or (21), Marcilly-sur-Tille, les Creux, on branch of *Populus* aff. *nigra*, 2 Sep. 2012, A. Gardiennet A.G. 12107 (WU 36920; ex-epitype culture CBS 142768 = BM; MBT377828).

*Other material examined*: Germany, Nordrhein-Westfalen, Münsterland, [Münster-] Nienberge; on wood of *Populus* sp. (originally given as *Quercus*). Dec. 1865, Th. Nitschke (B 70 0009349).

*Notes*: For synonyms, see Rappaz (1995). Concerning typification, Nitschke (1867) only cited material from Handorf on *Sarothamnus* in his protologue. In their list of type specimens of Nitschke deposited in B, Gerhardt and Hein (1979) mention two envelopes mounted on a sheet without a place or date on the envelopes. However, the holotype B 70 0009297 now only contains a single envelope with an asexual morph with hyaline conidia, i.e. no sexual morph is present. Therefore, epitypification became necessary. Rappaz (1995) selected B 70 0009349 as the lectotype, but that material was not cited in the protologue. It is, however, authentic material of *Valsa macrospora* (collected by Nitschke before publication), as both Nitschke and Rappaz considered it to be the fungus described in the protologue.

*Barrmaelia macrospora* is usually easy to identify due to its large and relatively narrow ascospores with one lighter coloured side. The inconspicuous germ slit was best visible in B 70 0009349 (Fig. 2t). Cannon (2015) provides a description of a slightly deviating British collection with larger, occasionally one-septate ascospores measuring (23.5–)26–29 × 7–8.5 μm, which may represent a distinct species.

**Fig. 3:**
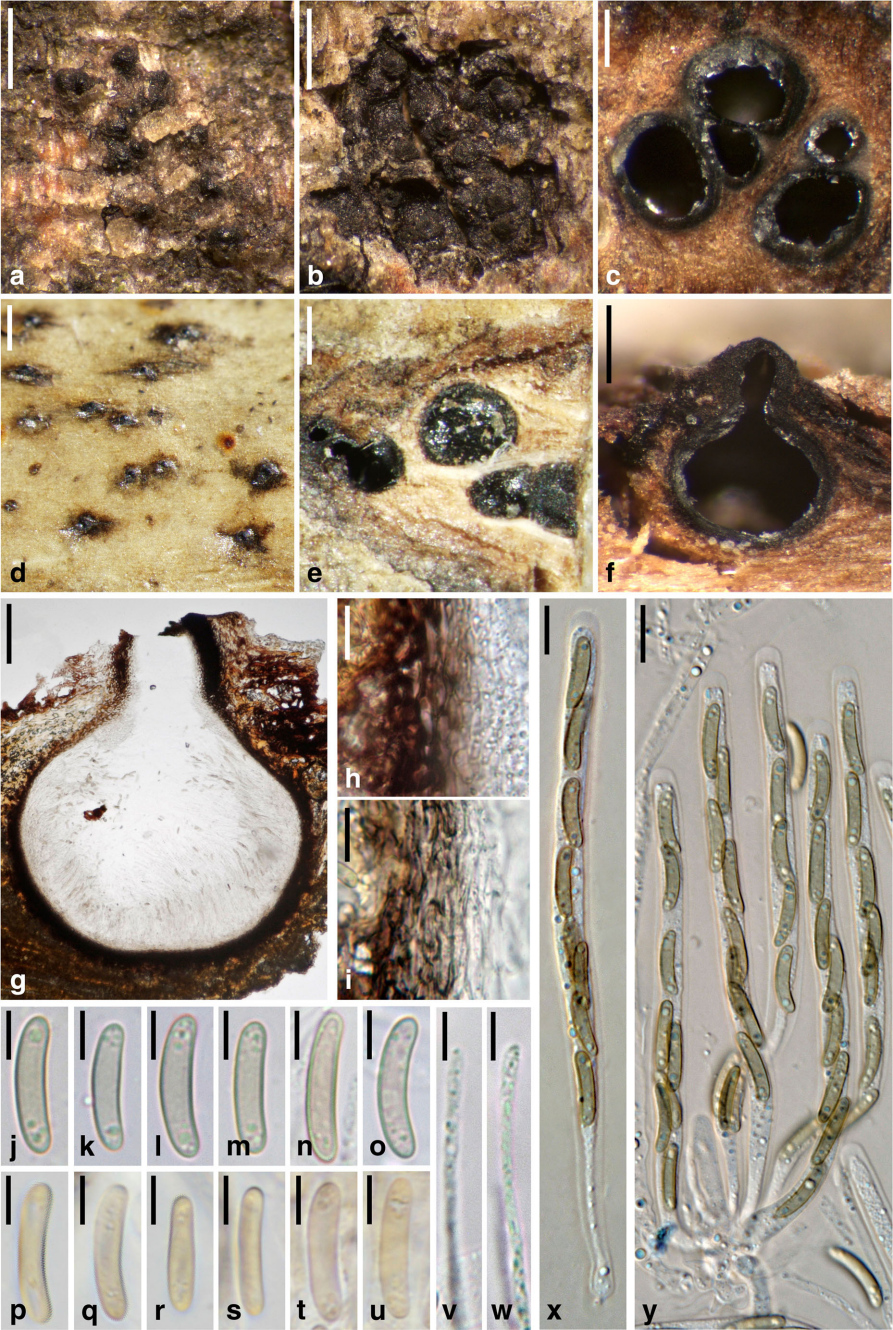
*Barrmaelia moravica* (**a**–**c**, **f**–**o**, **v**–**y**: WU 36924; **d**, **e**, **p**–**u**: W 1970-0024077, lectotype). **a**, **d** Ostioles protruding through the periderm. **b** Stroma beneath the periderm. **c**, **e** Perithecia in transverse section. **f**, **g** Perithecium in vertical section. **h**, **i** Vertical section of perithecial wall. **j**–**u** Ascospores. **v**, **w** Paraphyses apices. **x**, **y** Asci. All in water. Scale bars: **a**, **b**, **d** = 500 μm; **c** = 250 μm; **e** = 300 μm; **f** = 200 μm; **g** = 100 μm; **h**, **i**, **x**, **y** = 10 μm; **j**–**w** = 5 μm

***Barrmaelia moravica*** (Petr.) Rappaz, Mycol. Helv. 7(1): 134 (1995). Fig. 3.

*Basionym*. *Eutypa moravica* Petr., Ann. Mycol. 25(3/4): 224 (1927).

Stromata immersed in bark, covered by the periderm except for the ostiolar openings; in areas lacking periderm visible as black spots of up to 6 mm diam., not discolouring the periderm but sometimes blackening the bast around the perithecia. Ascomata perithecial, 300–700 μm (*n* = 15) diam., 200–500 mm high (*n* = 10), usually crowded to gregarious, rarely solitary, globose, ellipsoid to pyriform, contents whitish when immature, brown when mature. Ostioles conspicuous, papillate, often elongate, ostiolar pore rounded. Peridium 15–25 μm thick (*n* = 10), pseudoparenchymatous at the outer side and consisting of thick-walled dark brown cells, tending to be prosenchymatous, lighter coloured and thinner-walled at the inner side, partly filled with oil drops. Paraphyses numerous, narrowly thread-like, ca. 1–2 μm wide in the middle, filled with oil drops, tapering towards the apex. Asci 90–109 × 6–7 μm, spore part 63–100 μm, stipe 7–41 μm long (*n* = 20), cylindrical, containing eight obliquely uniseriate ascospores, with an inamyloid apical apparatus. Ascospores (12.3–)13.0–15.0(−16.3) × (2.3–)2.5–3.0(−3.3) μm, l/w = (4.2–)4.5–5.4(−6.0) (*n* = 60), one-celled, allantoid, without germ slit, light brown, filled with oil drops in the poles, smooth.

Colonies on CMD and MEA white; aerial hyphae abundant. No asexual morph observed.

*Habitat*: In bark of thin dead branches of *Salix caprea* attached to the tree.

*Distribution*: Europe (Austria, Czech Republic, Spain).

*Typification*. Lectotype of *Eutypa moravica* designated by Rappaz (1995): Czech Republic, Hranice (“Mährisch Weisskirchen“), Usti, on *Salix caprea*, Dec. 1925, F. Petrak (W 1970-0024077). Isotype: W 1978-0010895. Epitype of *Eutypa moravica*, here designated: Austria, Kärnten, Millstatt, Hinterdellach, on dead attached branch of *Salix caprea*, soc. *Platystomum compressum*, *Cyphellopsis* sp., *Capronia* sp., 3 Nov. 2015, W. Jaklitsch & H. Voglmayr (WU 36924; ex-epitype culture CBS 142769 = Cr1; MBT377829).

*Notes*: This species is well characterised by its light brown, allantoid and relatively small ascospores. *Barrmaelia rappazii* is superficially similar but differs morphologically mainly by larger and darker brown ascospores, and in having effused, black stromata with sparsely distributed perithecia. *Barrmaelia rhamnicola* also has allantoid ascospores but they are larger, filled with bigger oil drops and have a slightly darker colour, and it occurs on a different host.

**Fig. 4:**
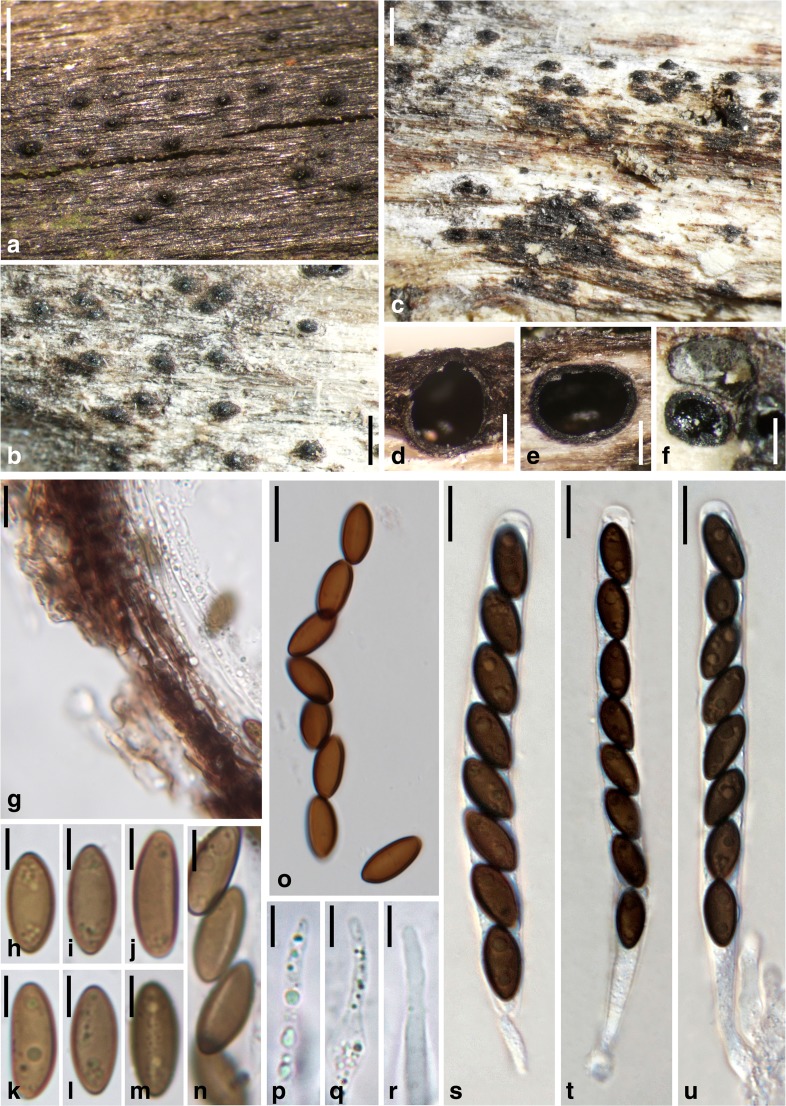
*Barrmaelia oxyacanthae* (**a**, **d**, **e**, **g**–**n**, **p**–**u**: WU 36925; **b**, **c**, **f**, **o**: PC 0706585, holotype). **a**–**c** Ostioles protruding through the blackened wood surface. **d** Perithecium in vertical section. **e**, **f** Perithecia in transverse section. **g** Vertical section of perithecial wall. **h**–**o** Ascospores (**o** in KOH + Melzer) with germ slits of spore-length (**j**–**n**). **p**–**r** Paraphyses apices. **s**–**u** Asci. All in water, except where noted. Scale bars: **a**–**c** = 500 μm; **d**, **f** = 250 μm; **e** = 350 μm; **g**, **o**, **s**–**u** = 10 μm; **h**–**n**, **p**–**r** = 5 μm

***Barrmaelia oxyacanthae*** (Mont.) Rappaz, Mycol. Helv. 7(1): 137 (1995). Fig. 4.

*Basionym*. *Sphaeria oxyacanthae* Mont., in Castagne, Suppl. Cat. Pl. Mars.: 48 (1851).

For synonyms, see Rappaz (1995).

Stromata discolouring the wood surface grey to black; wood usually showing no or only slight discolouration around the ascomata. Ascomata perithecial, 300–700 μm wide (*n* = 15), 300–500 μm high (*n* = 15), often closely spaced and arranged in lines, subglobose, ellipsoid to pyriform. Ostiolar necks with circular outline, ostioles shiny and slightly raised. Peridium 15–20 μm thick (*n* = 15), pseudoparenchymatous at the outer side and consisting of moderately thick-walled, dark brown cells, prosenchymatous, lighter coloured and thinner-walled at the inner side, partly filled with oil drops. Paraphyses numerous, 2–3.5 μm wide in the middle, filled with oil drops, slightly tapering towards the apex, obtuse. Asci 98–130 × 8–9 μm, spore part 73–100 μm long, stipe 15–34 μm long (*n* = 20), cylindrical, containing eight obliquely uniseriate ascospores, with an inamyloid apical apparatus. Ascospores (11.5–)12.3–14.2(−16.2) × (4.6–)5.3–6.3(−7.5) μm, l/w = (1.9–)2.1–2.5(−3.2) (*n* = 151), one-celled, ellipsoid, slightly inequilaterally, with a straight germ slit of spore-length (sometimes slightly shorter), brown to dark brown, filled with several small oil drops in the poles, smooth.

Colonies on CMD and MEA white; aerial hyphae abundant. No asexual morph observed.

*Habitat*: In wood of twigs and branches of various hardwoods.

*Distribution*: Widespread (Africa, Asia, Europe and North America); for details, see Cannon and Minter (2013).

*Holotype*: France, place and date unknown, in branches of *Crataegus oxyacantha*, soc. *Sphaeria lata* var. *corticalis*, L. Castagne (PC 0706585 ex herb. C. Montagne).

*Other material examined*: Austria, Steiermark, Deutschlandsberg, Koralpe, near the parking place of the walking path to the Grünanger- and Bärentalhütte; 15°00′52″E 46°49′37″N, on dead attached branches of *Salix* cf. *caprea*, 6 May 2016, G. Friebes (WU 36925; culture CBS 142770 = BO); Schadminger Tauern: Kleinsölk-Obertal, Schwarzensee, 1163 m, on *Salix* sp., 18 Sep. 1991, Ch. Scheuer 2897 (GZU 000317705). Germany, Sachsen, Königstein, on dead branches of *Salix purpurea*, Oct. 1880 and Apr. 1881, W. Krieger (GZU 000317701; as *Anthostoma schmidtii*); Schkeuditz, on dead branches of *Fraxinus excelsior*, spring 1874, G. Winter (GZU 000317700; as *Anthostoma schmidtii*). Italy, Venetia, Treviso, Selva, on decorticated dead branches of *Castanea vesca*, autumn 1873, P.A. Saccardo (GZU 000317702; as *Anthostoma schmidtii*). USA, South Dakota, Mellette, in a glacial valley, on branches of *Fraxinus* sp., Aug. 1950, F. Petrak (GZU 000317704; as *Anthostoma melanotes*); same data, 9 Aug. 1950, F. Petrak (GZU 000317703; as *Anthostoma melanotes*).

*Notes*: For synonyms, see Rappaz (1995). He found a libertella-like asexual morph in pure culture. Rappaz (1995) recognised three groups based on ascospore size within his broad concept of *B. oxyacanthae*. The first group with the smallest ascospores (“mean length between 12.5 and 13”) contains the type of *B. oxyacanthae* and agrees very well with GZU 000317702, whose mean length of 12.6 μm (*n* = 30) corresponds exactly with our measurements of the type collection. The sequenced collection WU 36925 has a mean length of 13.3 μm (*n* = 31) and, thus, appears to be an intermediate between the first and second group, the latter of which has a “mean length between 13.5–14”. The group with the longest ascospores (“between 14.5–15”) is said to mostly contain material on *Salix*. Of the three collections studied on this substrate, WU 36925 belongs to either the first or second group (see above), whereas GZU 000317705 falls in the second group (mean length 13.6 μm, *n* = 30) and GZU 000317701 best fits in the third group (mean length 14.3 μm, *n* = 30). GZU 000317700 does not contain mature ascomata. GZU 000317704 and GZU 000317703 from South Dakota (USA) have mean lengths of 16.2 and 19.6 (*n* = 30), respectively; thus, they likely represent different, probably undescribed, species.

*Barrmaelia oxyacanthae* differs from other *Barrmaelia* species in its relatively dark brown, ellipsoid ascospores with a well-visible germ slit. It is most similar to *B. pseudobombarda*, which has narrower ascospores (Rappaz 1995; Mathiassen et al. 2015). Cannon and Minter (2013) give a morphological description and illustrations of *B. oxyacanthae* and details on its ecology and distribution.

**Fig. 5:**
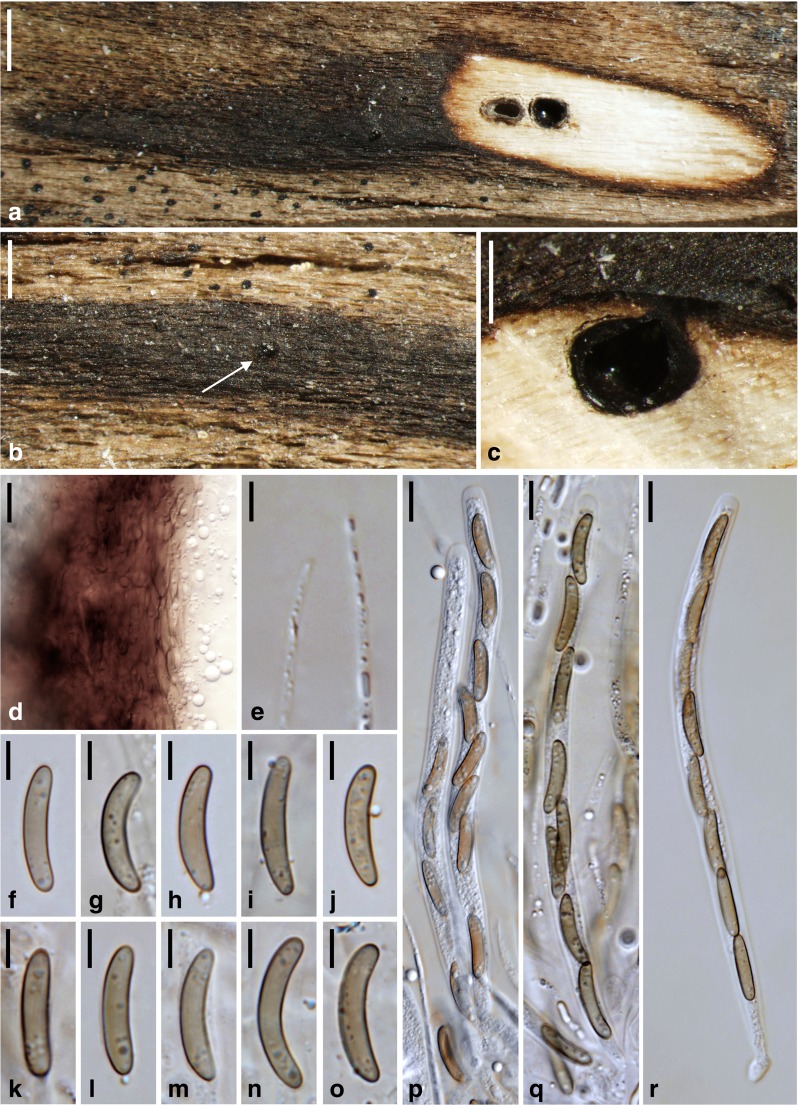
*Barrmaelia rappazii*, holotype (WU 36926). **a** Stroma with perithecia in transverse section. **b** Stroma (*arrow* denoting ostiole). **c** Perithecium in vertical section. **d** Vertical section of perithecial wall. **e** Paraphyses apices. **f**–**o** Ascospores. **p**–**r** Asci. All in water. Scale bars: **a** = 1 mm; **b**, **c** = 500 μm; **d**, **p**–**r** = 10 μm; **e**–**o** = 5 μm

***Barrmaelia rappazii*** Jaklitsch, Friebes & Voglmayr, sp. nov. Fig. 5.

MycoBank no.: MB 822043

*Etymology*: In honour of F. Rappaz, who established the genus *Barrmaelia*.

Stromata discolouring the wood surface grey to black in areas extending up to 6 × 0.6 cm; wood internally either nearly white between ascomata or darkened in patches. Ascomata perithecial, (450–)560–795(−900) μm (*n* = 14) diam., (420–)480–635(−660) μm (*n* = 9) high, sparsely distributed within the stromata and distantly spaced, immersed, depressed globose to ellipsoid. Ostioles forming minute, shiny black, rounded papillae above the wood surface. Peridium 20–45 μm thick (*n* = 7), pseudo- to prosenchymatous, cells moderately thick-walled and encrusted with brown material. Paraphyses up to 3.2 μm wide in the lower part, tapering, ca. 1 μm wide at the apex, filled with numerous oil drops when vital. Asci 117–158 × 5.8–8.5 μm, spore part 95–136 μm long, stipe 11.5–29.5 μm long (*n* = 11), cylindrical, containing eight uniseriate ascospores, with an inamyloid apical apparatus. Ascospores (12.8–)15.5–18.0(−19.5) × (2.8–)3.0–3.5(−3.8) μm, l/w = (3.8–)4.5–5.7(−6.5) (*n* = 39), one-celled, allantoid, brown, without germ slit, filled with few small oil drops, smooth.

Colonies on CMD and MEA white; aerial hyphae abundant. No asexual morph observed.

*Habitat*: In wood of twigs and branches of *Populus tremula*.

*Distribution*: Europe, only known from the type location in Norway.

*Holotype*: Norway, Stange, Hedmark, Rotlia Naturreservat, 7.5 km S Stange Kirke, on decorticated wood of *Populus tremula*, soc. *Platystomum compressum*, 30 Nov. 2015, P. Vetlesen PV-R221 (WU 36926; ex-holotype culture CBS 142771 = Cr2).

*Other material examined*: USA, North Dakota, Nylands Grove, on *Populus deltoides*, 29 Mar. 1914, J.F. Brenckle (W 1978-0018347, as *Anthostoma flavoviride*).

*Notes*: *Barrmaelia rappazii* can be recognised by its allantoid, brown and relatively narrow ascospores without a germ slit, and its black stromata with sparsely distributed ascomata. Morphological differences to the most similar species, *B. moravica*, are given there. *Barrmaelia rhamnicola* is another species with allantoid ascospores without a germ slit but they contain larger oil drops and are somewhat longer and wider.

Rappaz (1995) mentions a collection (W 1978-0018347) similar to *B. moravica* but growing on *Populus* and having larger and darker ascospores, thus apparently resembling *B. rappazii*. However, the examination of this collection revealed it to be a different species with shorter and wider ascospores measuring (12.0–)14.0–16.0(−16.8) × (3.3–)3.7–4.5(−5.0) μm, l/w = (2.5–)3.2–4.3(−4.9) (*n* = 30), as was already indicated by the congruent measurements given in Rappaz (1995). In the absence of sequence data, we currently refrain from describing it as a new species.

**Fig. 6:**
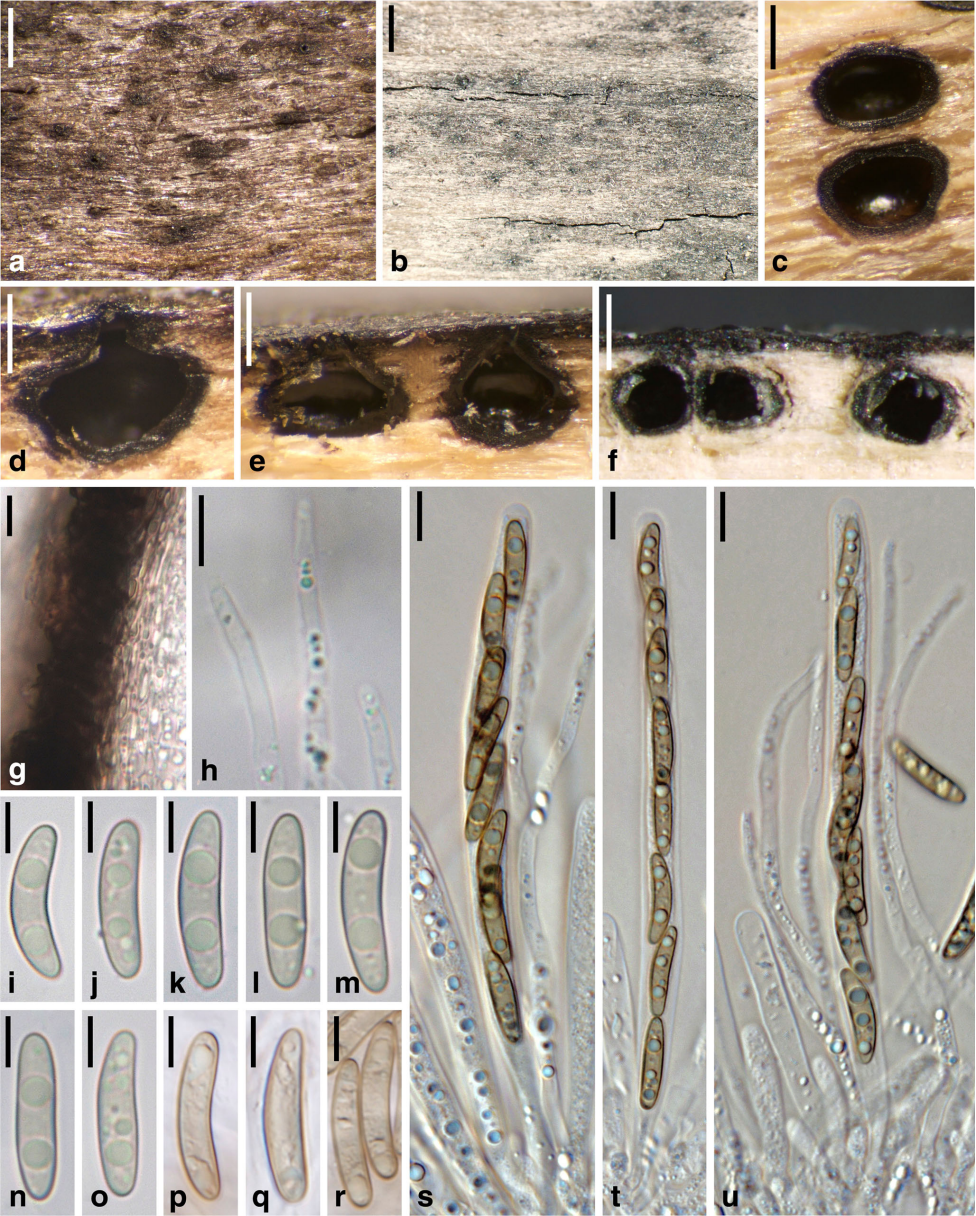
*Barrmaelia rhamnicola* (**a**, **c**, **e**, **g**–**l**, **s**–**u**: WU 36927; **d**, **m**–**o**: WU 36928, epitype; **b**, **f**, **p**–**r**: F. Rappaz no 890611-2, LAU, holotype). **a**, **b** Ostioles protruding through the blackened wood surface. **c** Perithecia in transverse section. **d**–**f** Perithecia in vertical section. **g** Vertical section of perithecial wall. **h** Paraphyses apices. **i**–**r** Ascospores. **s**–**u** Asci (with paraphyses in **u**). All in water. Scale bars: **a** = 500 μm; **b** = 1 mm; **c** = 250 μm; **d** = 150 μm; **e** = 200 μm; **f** = 300 μm; **g**, **h**, **s**–**u** = 10 μm; **i**–**r** = 5 μm

***Barrmaelia rhamnicola*** Rappaz, Mycol. Helv. 7(1): 130 (1995). Fig. 6.

Stromata discolouring the wood surface grey to black; wood showing no discolouration among ascomata. Ascomata perithecial, 200–500 μm wide (*n* = 15), 200–400 μm high (*n* = 10), usually gregarious but separate, rarely two in contact, immersed, outline in vertical section elliptic to broadly pyriform, in horizontal section broadly elliptic to round, contents whitish when immature, brown when mature. Ostiolar area often somewhat elongate but ostiolar necks with circular outline, ostioles rarely shiny and slightly raised. Peridium 20–25 μm thick (*n* = 15), pseudoparenchymatous at the outer side and consisting of thick-walled and dark brown cells, prosenchymatous, lighter coloured and thinner-walled at the inner side, partly filled with oil drops. Paraphyses numerous, 2–3 μm wide in the middle, filled with oil drops, slightly tapering towards the apex, obtuse. Asci 112–147 × 7–8 μm, spore part 81–110 μm long, stipe 14–32 μm long (*n* = 20), cylindrical, containing eight uni- or biseriate ascospores, with an inamyloid apical apparatus. Ascospores (14.8–)16.3–19.3(−21.3) × (3.3–)3.8–4.5(−5.0) μm, l/w = (3.4–)4.0–4.8(−5.3) (*n* = 90), smooth, one-celled, no sheath or appendages observed, without germ slit, usually slightly allantoid, light brown, filled with numerous oil drops (or two oil drops when dead).

Colonies on CMD and MEA white; aerial hyphae abundant. No asexual morph observed.

*Habitat*: In wood of decorticated dead twigs and branches of *Rhamnus alpina*.

*Distribution*: Europe (France, Spain, Switzerland).

*Typification*. Holotype: Switzerland, Vaud, les Rochers-de-Naye, Sautaudon, on *Rhamnus alpina*, June 1989, F. Rappaz no 890611-2 (LAU). Epitype of *Barrmaelia rhamnicola*, here designated: France, Côte-d’Or (21), Etaules, le Plain d’Avaux, on dead branch of *Rhamnus alpina*, 21 Jan. 2016, A. Gardiennet A.G. 16009 (WU 36927; ex-epitype culture CBS 142772 = BR; MBT377830).

*Other specimens examined*: France, Côte-d’Or (21), Chenôve, le Plateau, on dead branch of *Rhamnus alpina*, 26 Jan. 2016, A. Gardiennet A.G. 16011 (WU 36928; culture BR1). Spain, Asturias, Somiedo, way up to Altu de la Farrapona, Carboneo, ca. 1400 m s.m., on decorticated branches of *Rhamnus alpina*, 9 Jun. 2017, J. Fournier J.F. 17014 (WU 35984).

*Notes*: *Barrmaelia rhamnicola* is distinguished from other species of the genus by the often slightly curved, relatively large ascospores, which are filled with conspicuous oil drops and lack a germ slit. For comparison with the other allantoid-spored species without germ slit, see notes under *B. moravica* and *B. rappazii*. Rappaz (1995) observed a libertella-like asexual morph in pure cultures.

***Entosordaria*** (Sacc.) Höhn., Sber. Akad. Wiss. Wien, Math.-naturw. Kl., Abt. 1129: 167 (1920), emend.

*Synonym*. *Stereosphaeria* Kirschst., Ann. Mycol. 37(1/2): 96 (1939).

*Type species*: ***Entosordaria perfidiosa*** (De Not.) Höhn.

Ascomata perithecial, scattered, immersed to erumpent, depressed globose to ellipsoid, circular in transverse section. Peridium brown. Hamathecium of apically free, thin, sparsely branched paraphyses. Asci unitunicate, cylindrical, with uniseriate ascospores; apex inamyloid without distinct ring or amyloid with a discoid ring. Ascospores two-celled with septum near one end, the small cell hyaline, the large cell dark brown and with an apical germ apparatus consisting of radial slits. Asexual morph unknown.

*Notes*: *Entosordaria* was first described as a subgenus of *Anthostomella* (Saccardo 1882) and raised to the generic rank by Höhnel (1920), with *E. perfidiosa* as the generic type. Eriksson (1966) outlined the fundamental morphological differences from *Anthostomella*, i.e. inamyloid asci and dorsiventrally flattened ascospores with an apical germ apparatus consisting of radiating slits. He confined *Entosordaria* to the generic type and removed the genus from the *Xylariaceae*. Later, he (in Eriksson and Hawksworth 1986) argued that *Stereosphaeria* is the valid generic name to be used, considering *Entosordaria* (Sacc.) Höhn. to be a younger heterotypic homonym of *Entosordaria* Speg. However, *Entosordaria* Speg. has not been validly described according to ICN Art. 38.1, as Spegazzini (1910) neither provided a diagnosis nor a reference to a previous valid description. Therefore, *Entosordaria* (Sacc.) Höhn. remains available and, based on priority, is the generic name to be used.

Barr (1989) classified *E. perfidiosa* in *Clypeosphaeria*, based on similarities of their ascospores, apical ascus apparatus, ascomata, clypei and peridium structure. However, molecular phylogenies do not support a close relationship, as the generic type, *Clypeosphaeria mamillana*, is placed in Xylariaceae s. str. with high support (Fig. 1).

With the addition of the closely related *E. quercina*, the genus *Entosordaria* also includes a species with an amyloid ascus ring, which shows that this character is not suitable for generic classification within Xylariales.

**Fig. 7:**
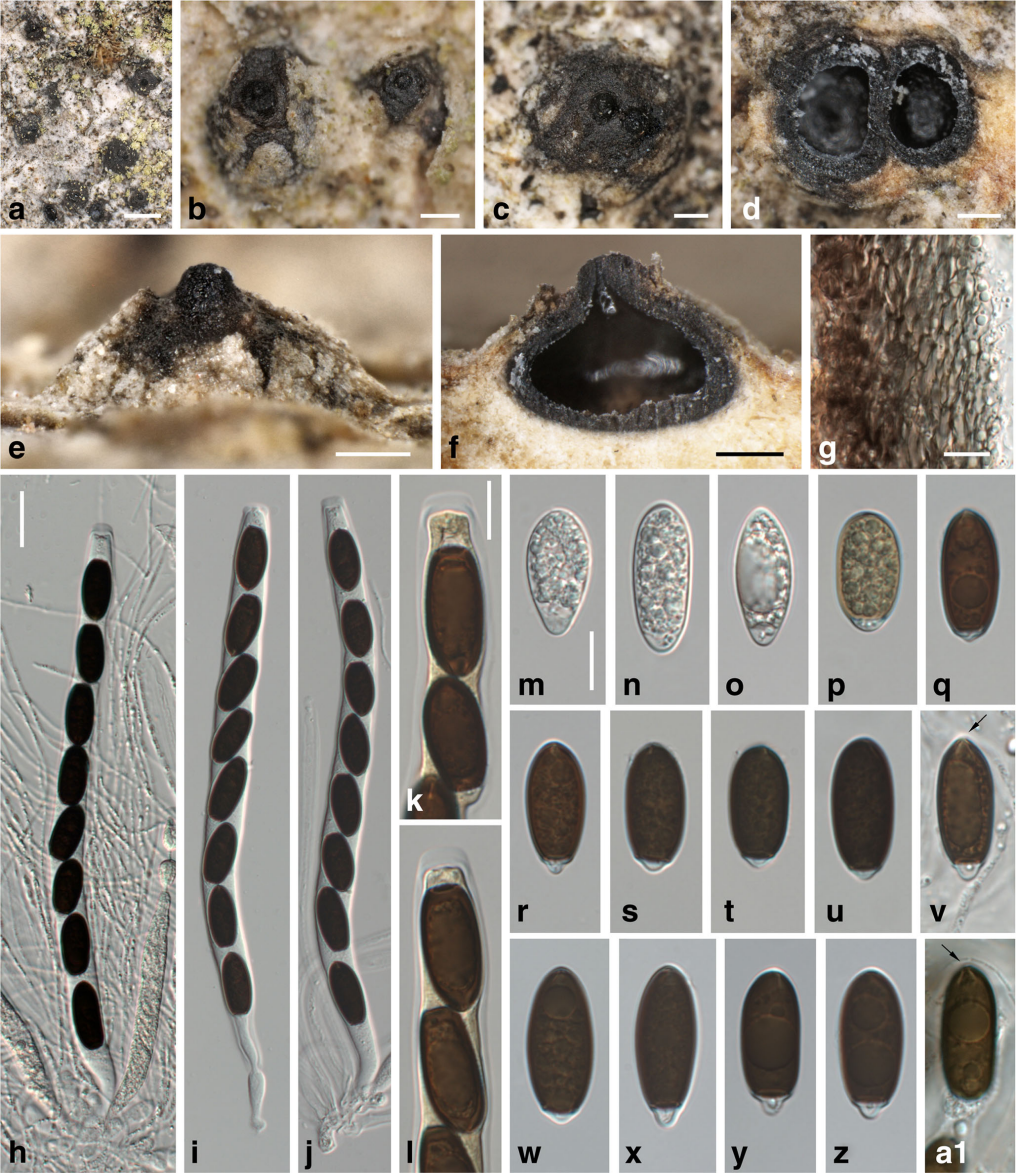
*Entosordaria perfidiosa* (**a**–**n**, **p**–**v**: WU 35981, epitype; **o**, **w**–**a1**: WU 35982). **a**–**c** Erumpent ascomata with apical papilla in face view (two fused ascomata in **c**). **d** Two ascomata in transverse section. **e** Ascoma with apical papilla in side view. **f** Ascoma in vertical section. **g** Transverse section of perithecial wall (in 3% KOH). **h**–**j** Asci (in 3% KOH; with paraphyses in **h**). **k**, **l** Ascus apices (in 3% KOH + Lugol). **m**–**a1** Ascospores (**m**–**p** immature; **v** in 3% KOH); the *arrows* denot﻿﻿e﻿ radial slits of the apical germ apparatus (**v**, **a1**). All in water, except where noted. Scale bars: **a** = 1 mm; **b**–**f** = 200 μm; **g**, **k**–**a1** = 10 μm; **h**–**j** = 20 μm

***Entosordaria perfidiosa*** (De Not.) Höhn., Sber. Akad. Wiss. Wien, Math.-naturw. Kl., Abt. 1129: 166 (1920). Fig. 7.

*Basionym*. *Sordaria perfidiosa* De Not., Comm. Soc. crittog. Ital. 2(fasc. 3): 481 (1867).

For synonyms, see Barr (1989).

Ascomata perithecial, scattered, solitary or in groups, partly immersed to erumpent, depressed globose to ellipsoid, circular in transverse section, 400–800 μm diam., with a distinct central apical papilla 120–200 μm wide. Peridium 20–40 μm wide, brown, pseudoparenchymatous, of dark brown isodiametric to elongate cells 2–12 μm diam. Paraphyses numerous, sparsely branched, 1–2 μm wide. Asci in 3% KOH (185–)200–220(−225) × (11–)12–14(−15) μm (*n* = 27), unitunicate, cylindrical, with a short stipe, with eight (partly obliquely) uniseriate ascospores, with an inamyloid apical apparatus, no apical ring seen. Ascospores (20.5–)21.8–25.8(−29.5) × (8.7–)10.0–11.2(−12.0) μm, l/w = (1.8–)2.1–2.5(−2.7) (*n* = 71), ellipsoid, with subacute to rounded ends, two-celled with septum near one end, the small cell hyaline, the large cell dark brown at maturity and with an apical germ apparatus consisting of radial slits, multiguttulate when young, at maturity often with a large and several small guttules.

Colonies on CMD and MEA white; aerial hyphae abundant. No asexual morph observed.

*Habitat*: In bark of old trunks of living *Acer pseudoplatanus*.

*Distribution*: Europe.

*Typification*. Lectotype of *Sordaria perfidiosa*, here designated: Italy, Riva, Corteccia dell’*Acer pseudoplatanus*, 30. Oct. 1863, Ab. Carestia, no. 413 (RO; MBT377831). Syntype: Riva, Sulla corteccia dell’*Acer pseudoplatanus*, 22 Apr. 1858, Ab. Carestia, no. 222 (RO). Epitype of *Sordaria perfidiosa*, here designated: Austria, Kärnten, St. Margareten im Rosental, at Brici (“Writze”), on bark of *Acer pseudoplatanus*, 10 Apr. 2016, H. Voglmayr & W. Jaklitsch (WU 35981; ex-epitype culture CBS 142773 = EPE; MBT377832).

*Other material examined*: Germany, Baden-Württemberg, Hornberg, on bark of *Acer pseudoplatanus*, Dec. 2015, B. Wergen (WU 35982; culture BW3). France, Hautes-Alpes (05), Vallouise-Pelvoux, Ailefroide, on ﻿bark of living trunk of *Acer pseudoplatanus*, soc. *Decaisnella mesascium*, 28 Jul. 2017, A. Gardiennet A.G. 17056.

*Notes*: *Entosordaria perfidiosa* is well characterised by the ascospores with an apical germ apparatus consisting of radiating slits in combination with inamyloid asci and its growth on bark of old *Acer pseudoplatanus* trees. It has been classified in *Clypeosphaeria* by Barr (1989); however, it is only distantly related with *C. mamillana*, the generic type (see Fig. 1). For comparison with *E. quercina*, see below.

Two syntypes of *Sphaeria perfidiosa* are present at RO, which were studied in detail by O. Eriksson (see Eriksson and Hawksworth 1986), but he did not select a lectotype. Type specimens at RO are no longer sent out for study, but detailed photographic documentation of the two syntypes was generously provided by Mrs. A. Millozza (pers. comm.). Based on the abundance of ascomata, we select no. 413 as the lectotype. For nomenclatural stability, a recent Austrian collection for which a culture and DNA sequences are available is designated as the epitype.

**Fig. 8:**
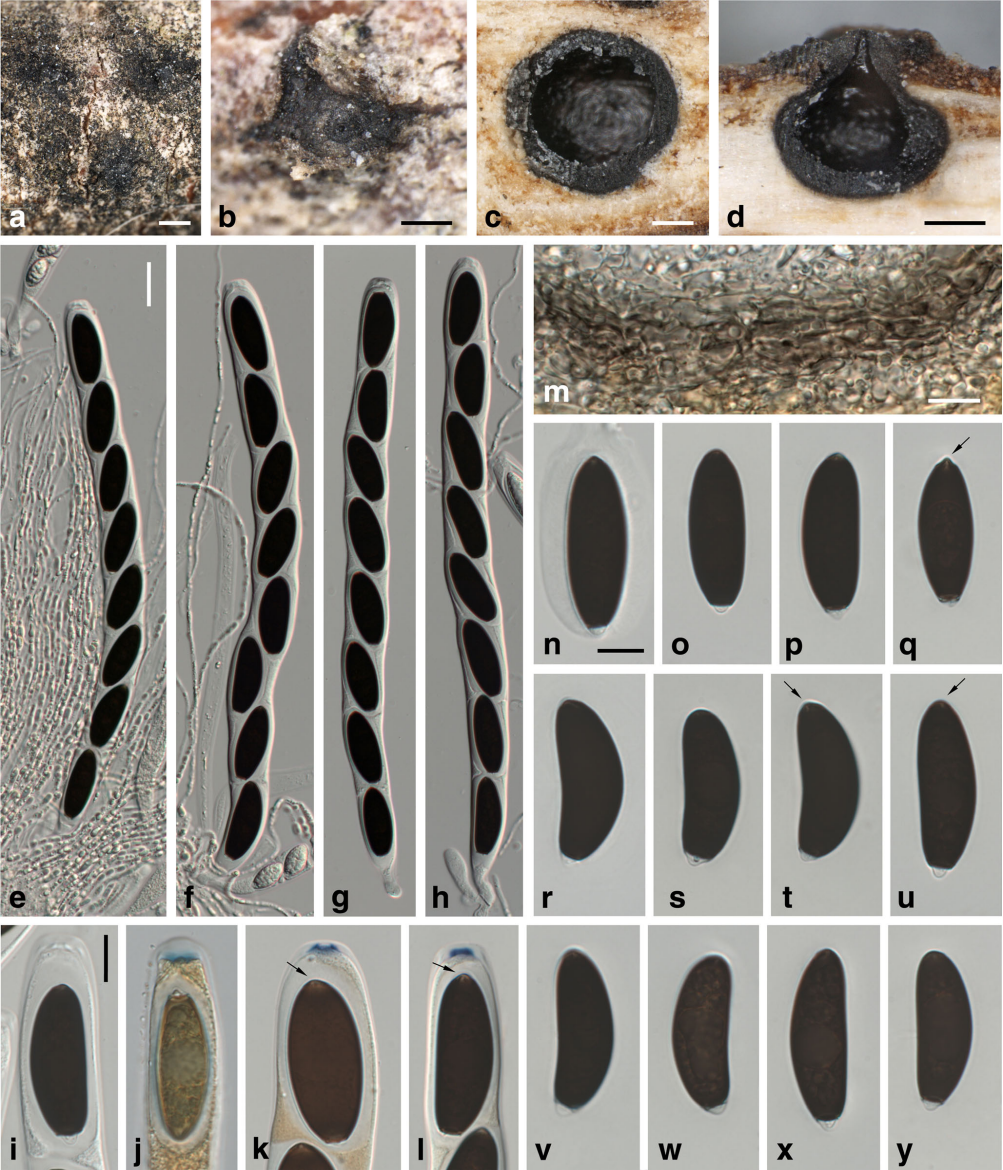
*Entosordaria quercina*, holotype (WU 35983). **a**, **b** Ascomata immersed in bark in face view. **c** Ascoma in transverse section. **d** Ascoma in vertical section. **e**–**h** Asci (with paraphyses in **e**, **f**). **i**–**l** Ascus apices (**j** in Lugol; **k**, **l** in 3% KOH + Lugol; note the gelatinous sheath surrounding the ascospores, the *arrows* in **k** and **l** denote the radial slits of the apical germ apparatus of the ascospore). **m** Transverse section of perithecial wall. **n**–**y** Ascospores; the *arrows* denote the short radial slits of the apical germ apparatus. All in 3% KOH, except where noted. Scale bars: **a** = 500 μm; **b**–**d** = 200 μm; **e**–**h** = 20 μm; **i**–**y** = 10 μm

***Entosordaria quercina*** Voglmayr & Jaklitsch, sp. nov. Fig. 8.

MycoBank no.: MB 822044

*Etymology*: Referring to the host genus *Quercus*.

Ascomata perithecial, scattered, solitary, immersed below bark and raising it, depressed globose to ellipsoid, circular in transverse section, 400–800 μm diam., without an apical papilla, ostiole not to slightly protruding above cortex. Peridium 20–30 μm wide, brown, pseudoparenchymatous, of dark brown isodiametric to elongate cells 3–11 μm diam. Paraphyses numerous, sparsely branched, 1.5–2.5 μm wide. Asci in 3% KOH (258–)270–293(−310) × (17–)18.5–21.5(−22) μm (*n* = 17), unitunicate, cylindrical, with a short stipe, with eight (partly obliquely) uniseriate ascospores, ascus apex containing a discoid amyloid apical ring 5.3–6.8 × 1.3–1.8 μm (*n* = 15; in 3% KOH + Lugol). Ascospores (31–)34–38(−40) × 12–13.5(−16) μm, l/w = (2.4–)2.7–3.0(−3.2) (*n* = 50), ellipsoid to allantoid, two-celled with septum near one end, the small cell hyaline, the large cell dark brown at maturity and with an apical germ apparatus consisting of radial slits, multiguttulate when young, at maturity often with a large and several small guttules, surrounded by a gelatinous sheath quickly dissolving in water.

Colonies on CMD white, on MEA a reddish and yellowish pigment developing; aerial hyphae abundant. No asexual morph observed.

*Habitat*: In bark of dead twigs of *Quercus coccifera*.

*Distribution*: Only known from the type locality in Crete (Greece).

*Holotype*: Greece, Crete, Chania, Omalos, 920 m s.m., 35.37° N, 23.897° E, in bark of *Quercus coccifera*, 5 June 2015, H. Voglmayr & W. Jaklitsch (WU 35983; ex-holotype culture CBS 142774 = RQ).

*Notes*: Ascospore morphology of *Entosordaria quercina* fits *E. perfidiosa*, from which it differs in an amyloid ascus ring, much larger ascospores and asci, immersed ascomata without a distinct apical papilla and the host. In addition, the ascospores of *E. quercina* are commonly allantoid. The radiating slits of the apical germ apparatus are shorter than in *E. perfidiosa*; thus, they are less distinct.

## Discussion

### Phylogenetic relationships and familial classification within Xylariaceae sensu lato

Our phylogenetic analyses are fully concordant with Wendt et al. (2017) in revealing Hypoxylaceae, Graphostromataceae and Xylariaceae sensu stricto as highly supported distinct lineages within the former Xylariaceae sensu lato, with Hypoxylaceae being placed basal to the rest (Fig. 1). The highly supported *Barrmaelia*–*Entosordaria* clade is also contained within the highly supported Xylariaceae sensu lato, but not affiliated with any of these families; a sister group relationship to Xylariaceae sensu stricto receives only moderate support (71%) in ML analyses and is unsupported in the MP analyses. Therefore, to be consistent with the new familial classification of Wendt et al. (2017), the *Barrmaelia*–*Entosordaria* clade is classified here within the new family Barrmaeliaceae.

Our data also demonstrate that the genus *Entosordaria* is phylogenetically distinct from *Clypeosphaeria*, disproving the generic concept of Barr (1989). In the phylogenetic analyses of Jaklitsch et al. (2016) based on ITS–LSU sequence data, the generic type *Clypeosphaeria mamillana* was contained within Xylariaceae sensu lato, but its closest relatives remained unclear due to the lack of internal backbone support. In our multigene analyses, the phylogenetic position of *Clypeosphaeria mamillana* is now resolved to belong to Xylariaceae sensu stricto, where it forms a highly supported basal clade together with *Anthostomelloides krabiensis*, the generic type of *Anthostomelloides* (Fig. 1). Although both species differ in their ascospore characters, they share a similar wedge-shaped amyloid apical apparatus (Jaklitsch et al. 2016; Tibpromma et al. 2017). The second species of *Anthostomelloides* included in our analyses, *A. forlicesenica*, is not revealed as being closely related to the generic type, but as a sister species to *Brunneiperidium gracilentum* with high to maximum support (Fig. 1), with which it shares a discoid amyloid apical apparatus. This discrepancy in phylogenetic placement compared to Daranagama et al. (2016) may be caused by their obviously erroneous LSU and *rpb2* sequences (see above), which were excluded from our analyses. This has been confirmed by an MP analysis of the matrix including the erroneous (pleosporalean) *rpb2* and LSU sequences of *A. forlicesenica*, which result in an unsupported phylogenetic position of the latter as sister to the *A. krabiensis*–*Clypeosphaeria* clade (not shown).

As, apart from the commonly sequenced ITS–LSU rDNA, few sequence data are available for most lineages of Xylariales, the phylogenetic position of many taxa of putative xylariaceous affinities remains unresolved (Wendt et al. 2017). Whereas the ITS–LSU sequences are useful for barcoding purposes, molecular phylogenies solely based on these markers commonly do not provide sufficient phylogenetic resolution, and backbone support of many deeper nodes is often low (e.g. Jaklitsch and Voglmayr 2012; Jaklitsch et al. 2014, 2016). Considering the substantial increase of phylogenetic resolution observed in the multigene analyses of Wendt et al. (2017) and the current study (Fig. 1), *rpb2* and *tub2* should be included as standard markers in future phylogenetic studies of Xylariales, in addition to the usually sequenced ITS–LSU rDNA.

### *Anthostomella* and *Anthostomella*-like genera

Recently, several investigations were published on *Anthostomella* (Daranagama et al. 2015, 2016; Tibpromma et al. 2017). In these publications, the genus *Anthostomella* was recognised to be polyphyletic, and several new genera and species were established.

Due to the lack of sequence data for *rpb2* and *tub2*, only a subset of these taxa could be incorporated in our analyses. However, for most new *Anthostomella*-like genera, at least the generic type could be included, and we believe that some of the results are conclusive and should encourage more detailed studies and a critical evaluation of the published data. There are some topological differences between our analyses and those of Daranagama et al. (2015, 2016), which may be caused by the inclusion of some obviously erroneous sequences in the latter (see the Results section above). In our analyses, *Pseudoanthostomella* and *Anthostomella* in the sense of Daranagama et al. (2016) were united in a highly supported clade clearly placed outside Xylariaceae sensu lato (Fig. 1), whereas in Daranagama et al. (2016), *Pseudoanthostomella* and *Anthostomella* formed separate clades (clades A and C in their fig. 2). However, they only included members of Xylariaceae sensu lato in their analyses, with a single distantly related sordariomycete, *Sordaria fimicola*, as the outgroup, and internal support of the tree backbone relevant for the topology of *Anthostomella*-like fungi was low or absent. In our ML analysis, the *Pseudoanthostomella*–*Anthostomella* clade is the sister group of a highly supported clade containing *Neoanthostomella viticola* and *Calceomyces lacunosus*, the latter representing a genus of uncertain affinities within Xylariales (Wendt et al. 2017). The monotypic genus *Alloanthostomella*, introduced by Daranagama et al. (2016) for *Anthostomella rubicola*, is not supported in our analyses, as it is placed within the highly supported *Anthostomella* clade (Fig. 1), a position which was also revealed in Daranagama et al. (2015).

Our phylogenies suggest that the *Pseudoanthostomella*–*Anthostomella* clade may represent a distinct family (Fig. 1). However, we refrain from formally establishing a new family because the generic type, *Anthostomella limitata*, has not been sequenced, and it is, as yet, unclear whether *Anthostomella* in the sense of Daranagama et al. (2015, 2016) phylogenetically includes the generic type. Therefore, the correct application and circumscription of *Anthostomella* remains uncertain until sequences of the generic type become available.

In our molecular phylogenetic analyses, the *Anthostomella*-like *Pyriformiascoma trilobatum* is placed within *Microdochium* with maximum support (Fig. 1). The sexual morphs of *Microdochium* have thin-walled, hyaline to pale brown, fusoid ascospores with commonly variable but more or less regular septation and asci with a distinct funnel-shaped amyloid apical ascus apparatus (Parkinson et al. 1981; Jaklitsch and Voglmayr 2012; Hernández-Restrepo et al. 2016). *Pyriformiascoma trilobatum* differs substantially from all known sexual morphs of *Microdochium* by two-celled, inequilateral, oblong-ellipsoid ascospores consisting of a large olivaceous-brown cell and a hyaline dwarf cell and by an indistinct inamyloid apical ascus apparatus (Daranagama et al. 2015). Considering that *Microdochium* is morphologically homogeneous, it is unlikely that *Pyriformiascoma* belongs there, and the sequences of the latter may, rather, originate from a *Microdochium* contaminant. The “conidia” illustrated for *P. trilobatum* in Daranagama et al. (2015) recall unicellular terminal chlamydospores which are known from *Microdochium* species (Hernández-Restrepo et al. 2016). *Pyriformiascoma trilobatum* should be re-sequenced from verified cultures to ascertain its phylogenetic position.

An evaluation of published sequences reveals that sequence data quality should be critically checked by BLAST searches and detailed inspection of alignments before inclusion into phylogenetic analyses. An indicator for problems in the sequence data used for phylogenetic analyses are exceptionally long branch lengths in phylograms like, for example, those seen for some clades in Daranagama et al. (2016). Marked topological differences between our analyses and those of previous publications (Daranagama et al. 2015, 2016), but also between the latter, may, at least partly, be due to the inclusion of obviously inaccurate, dubious or erroneous sequences which have been identified and removed from our matrix. These errors may cast general doubts on the accuracy of the sequences published for these species, and all markers should be re-sequenced from verified material to corroborate their phylogenetic affinities.

## Electronic supplementary material

Below is the link to the electronic supplementary material.

ESM 1 (PDF 11 kb)
