# Supplementary figures and images for: Barrmaelia and Entosordaria in Barrmaeliaceae (fam. nov., Xylariales) and critical notes on Anthostomella-like genera based on multigene phylogenies
Source: Mycol Prog. 2017 Aug 23;17(1):155–77. doi: 10.1007/s11557-017-1329-6 (PMC5801398; doi:10.1007/s11557-017-1329-6)

Strict consensus of 4 most parsimonious trees of score 32311

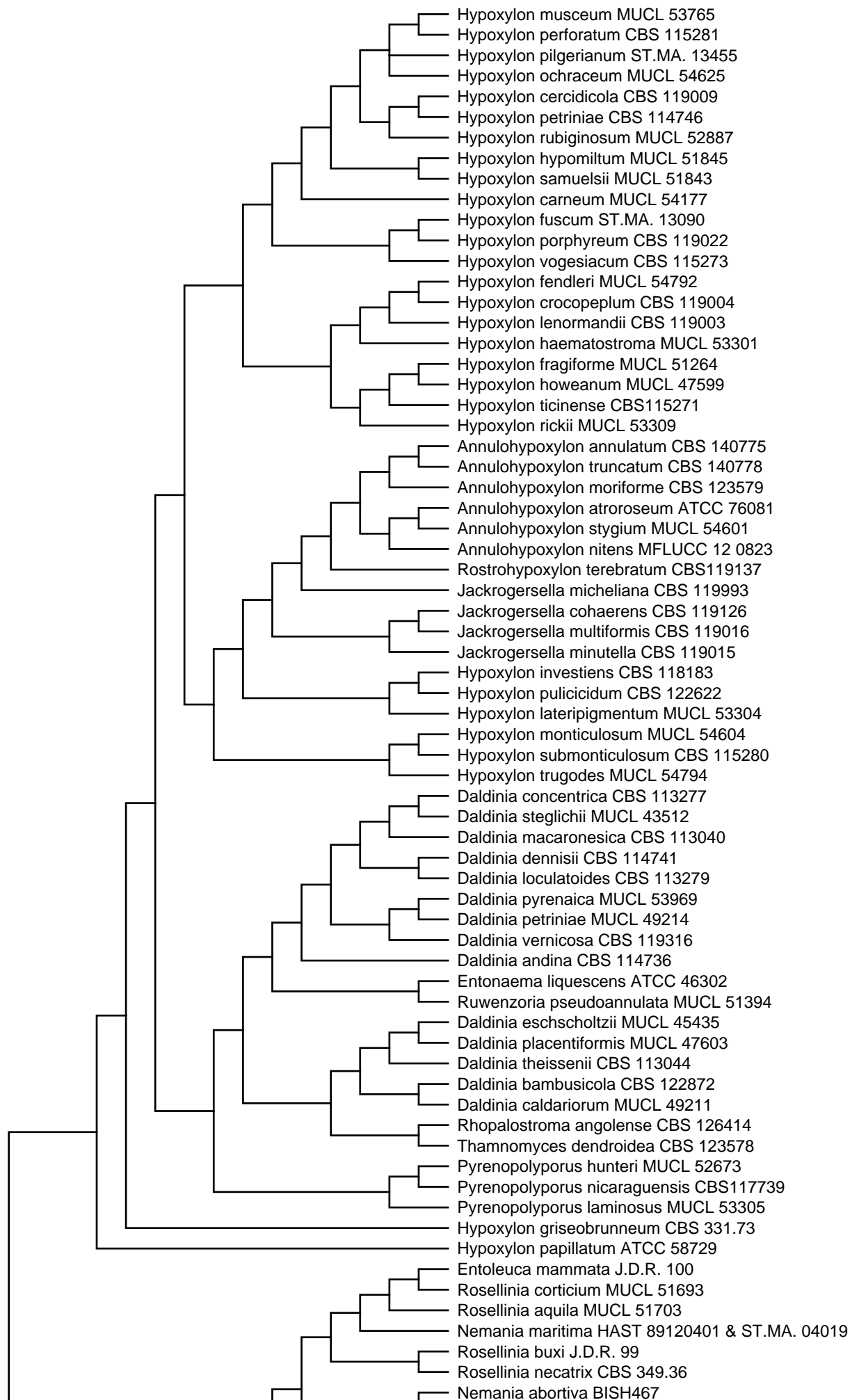

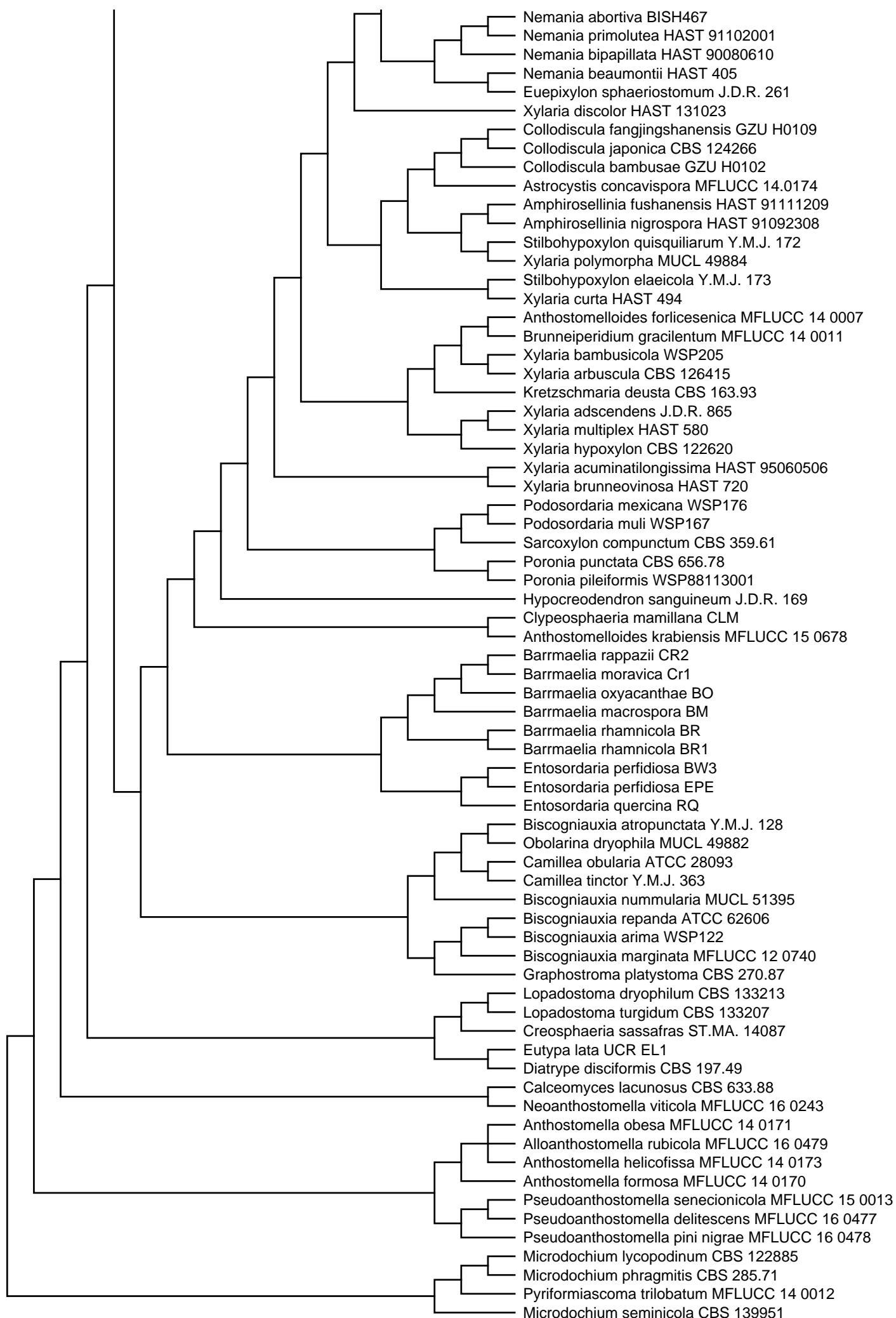

Supplement: Supplementary file 1 — (PDF 11 kb) [file 11557_2017_1329_MOESM1_ESM.pdf]
